# Impact of Ginger Straw on Cultivation and Quality of *Pleurotus geesteranus* and *Hericium erinaceus*

**DOI:** 10.3390/foods15050898

**Published:** 2026-03-05

**Authors:** Yan Zhang, Yihui Wang, Qingji Wang, Zheng Li, Zhuang Li

**Affiliations:** Shandong Provincial Key Laboratory of Agricultural Microbiology, College of Plant Protection, Shandong Agricultural University, Tai’an 271018, China; zhangyan6032@163.com (Y.Z.); wangyihui202601@163.com (Y.W.); ericwong@sdau.edu.cn (Q.W.)

**Keywords:** ginger straw, edible fungus, antioxidant characteristic, nutritional properties, biological efficiency

## Abstract

Against the backdrop of China’s booming edible fungi industry, shortages and price hikes of traditional cultivation substrates have emerged as critical bottlenecks. Meanwhile, the disposal of a large amount of ginger straw produced during the ginger cultivation process is also a major challenge. To address these issues, this study explored ginger straw as an alternative substrate for *Pleurotus geesteranus* and *Hericium erinaceus*, focusing on the optimization of substrate formulas and their effects on the nutritional quality of the fungi. Superior strains were first screened, after which the addition ratios of ginger straw (10–40%) were optimized. Commercial characteristics, nutritional components, and safety indicators of the fruiting bodies were determined, and a comprehensive quality evaluation was conducted using the membership function method. Results indicated that excellent strains of both fungi were selected: the optimal ginger straw addition ratio was 15–30% for *P. geesteranus* and 15% for *H. erinaceus*. Compared with the conventional cottonseed hull substrate, the optimized formulas significantly increased the biological efficiency (BE) by 9.08–27.1% for *P. geesteranus* and 9.16% for *H. erinaceus*. They also improved the contents of key nutrients (e.g., proteins and amino acids), enhanced total antioxidant capacity, and optimized the composition of flavor-contributing amino acids. This study offers a novel approach for the efficient utilization of ginger straw, provides technical and theoretical support for the low-cost and high-quality cultivation of edible fungi, and contributes positively to the development of ecological circular agriculture.

## 1. Introduction

Ginger (*Zingiber officinale Roscoe*), a plant belonging to the Zingiberaceae family, is cultivated primarily for its edible underground fleshy rhizome. Rich in essential nutrients and bioactive compounds, it exhibits a range of beneficial properties, including anti-inflammatory, analgesic, antioxidant, hypoglycemic, and lipid-lowering effects, as well as benefits for stomach and liver health [1,2]. With the continuous expansion of ginger cultivation, the resource utilization of ginger straw has gradually become a prominent issue. Large quantities of ginger straw have been indiscriminately discarded or burned, leading not only to resource wastage but also to a series of ecological and agricultural problems, such as environmental pollution and disease transmission. Studies show that ginger straw holds significant potential for utilization [3,4]. It not only contains crude protein and crude fat but also has low contents of neutral detergent fiber and acid detergent fiber. Meanwhile, ginger straw is abundant in soluble sugars, crude fiber, vitamins, trace elements, and ginger-specific bioactive compounds such as curcumin and gingerol [5]. Therefore, exploring scientific and efficient methods for straw utilization is of great practical significance for promoting the recovery of agricultural waste and alleviating ecological pressures. Among various edible fungus species, *P. geesteranus* (synonym: *Pleurotus pulmonarius* (Fr.) Quél.) and *H. erinaceus* have attracted considerable attention due to their excellent nutritional quality and market prospects. *P. geesteranus* is characterized by low fiber and high protein content and is rich in essential amino acids such as lysine, threonine, and leucine [6,7]. *H. erinaceus* contains abundant protein, fat, fiber, polysaccharides, amino acids, and unsaturated fatty acids. It exhibits various bioactive properties, including lowering cholesterol levels, enhancing immune function, delaying aging, and inhibiting cancer cell development. Notably, it has excellent therapeutic effects on digestive diseases, such as gastric ulcers and gastritis [8,9,10].

Agricultural straw, with its wide availability, low cost, and richness in carbon and nitrogen sources essential for fungus growth, serves as an ideal alternative substrate for cultivation [11,12,13]. Traditional cultivation formulas for *P. geesteranus* and *H. erinaceus* heavily rely on sawdust. Employing single-straw substrates, such as broad bean straw, peanut straw, or rapeseed straw, or mixed substrates combining corncob, rice straw, and corn straw, has been shown to enhance the growth, yield, and biological efficiency (BE) of *P. geesteranus* while improving its agronomic traits [14,15]. Substituting part of the sawdust with soybean straw, hemp straw, corn straw, buckwheat straw, or wheat straw can increase the yield of *H. erinaceus*. In particular, when hemp straw is added at up to 40%, the BE of *H. erinaceus* is significantly superior to that of conventional sawdust-based formulas [16,17]. Furthermore, waste materials have also been utilized in the cultivation of *H. erinaceus* [18].

Based on the above background, we employed ginger straw for the cultivation of *H. erinaceus* and *P. geesteranus* and investigated its effects on the agronomic traits and nutritional quality of these edible fungi. This research not only addressed the environmental and resource issues associated with ginger straw disposal but also could reduce the cultivation costs of edible fungi, offering a viable option for the development of efficient and eco-friendly circular agriculture.

## 2. Materials and Methods

### 2.1. Collection and Cultivation of Experimental Strains

This study involved the collection of two species of edible fungi, totaling 19 strains, including both widely cultivated and rare varieties in China. The specific names and sources of these strains are detailed in Appendix A, Table A1. The collected strains were activated by inoculation onto potato–dextrose–agar (PDA: peeled potato 200.0 g, glucose 20.0 g, agar 20.0 g, KH_2_PO_4_ 3.0 g, MgSO_4_•7H_2_O 1.5 g, made up to a final volume of 1 L with deionized water). Natural pH (autoclaved at 121 °C for 0.5 h) medium was cultured under 25 °C with a humidity of 40%.

### 2.2. Screening of Superior Strain

#### 2.2.1. Screening on Potato-Dextrose-Agar (PDA) Medium

The activated strains were inoculated onto PDA medium, with three replicates per strain, and incubated in the dark at 25 °C and 40% relative humidity. On the third day after inoculation, mycelial growth was evaluated according to the method described in Appendix A, Table A2.

#### 2.2.2. Preliminary Screening on Conventional Cottonseed Hull Medium In Situ

Conventional cottonseed hull substrate was used for preliminary screening. For *P. geesteranus*, the control substrate consisted of 80% cottonseed hull, 18% wheat bran, 1% gypsum, and 1% white sugar. For *H. erinaceus*, the control substrate consisted of 90% cottonseed hull, 8% wheat bran, 1% gypsum, and 1% calcium superphosphate.

Test tubes (25 × 250 mm) were filled with 18 g of substrate (approximately two-thirds of the tube volume) and autoclaved at 121 °C for 120 min. Two mycelial plugs (5 mm in diameter and 2 mm in thickness) were inoculated into each tube, followed by incubation in darkness at 25 °C and 40% relative humidity. A starting line was marked on the fifth day after inoculation, and a termination line on the fifteenth day to measure mycelial growth distance. Mycelial growth performance was evaluated according to Appendix A, Table A2.

### 2.3. Optimization, Screening, and Fruiting Test of Ginger Straw Substrate Formulations

Seven substrate formulations were prepared with ginger straw ratios ranging from 10% to 40% (at 5% intervals). For *P. geesteranus*, each formulation contained ginger straw, cottonseed hull, 18% wheat bran, 1% gypsum, and 1% white sugar. For *H. erinaceus*, each formulation contained ginger straw, cottonseed hull, 1% gypsum, and 1% calcium superphosphate. Corresponding conventional cottonseed hull substrates were used as controls. All substrates were autoclaved at 121 °C, 0.12–0.14 MPa for 3 h. High-temperature-resistant polypropylene bags (15 × 27 cm) were filled with the abovementioned substrates. Each treatment included 10 replicate bags per strain, which were incubated at 25 °C with 60–70% relative humidity until mycelium fully colonized the bags. Fruiting management was then performed at 23–26 °C, 87–93% relative humidity, and 700–1000 ppm CO_2_ concentration.

### 2.4. Determination of Fruiting Body Traits

The total yield across two harvest flushes (22–40 days) was recorded, and the BE was calculated. Fresh samples of fruiting bodies were measured for cap thickness, cap diameter, and stipe length. The moisture content was determined using the direct drying method. Biological efficiency (%) = (Fresh weight of fruiting bodies/Dry weight of substrate) × 100%.

### 2.5. Determination and Assessment of Nutrient Components in Fruiting Bodies

The methods for nutrient determination were consistent with those employed in our previous studies [19]. Fresh fruiting bodies were air-dried in a ventilated area for 2 days, followed by drying in an Electrothermal blowing dry box (Model GZX-9146MBE, Shanghai Boxun Medical Biological Instrument Corp., Shanghai, China) at 50 °C until a constant weight was achieved. The dried samples were then ground using a high-speed grinder (Model FW100, Tianjin Tester Instrument Co., Ltd., Tianjin, China) and sieved through a 60-mesh sieve.

Nutrient components were determined in accordance with the following Chinese National Standards: GB 5009.5-2016 (Determination of Protein in Foods—Kjeldahl Method) [20], GB 5009.6-2016 (Determination of Fat in Foods—Soxhlet Extraction Method) [21], GB/T 15672-2009 (Determination of Total Sugar in Edible Fungi) [22], GB 5009.7-2016 (Determination of Reducing Sugar in Foods—Direct Titration Method) [23], GB 5009.124-2016 (National Food Safety Standard—Determination of Amino Acids in Foods) [24], GB 5009.268-2016 (National Food Safety Standard—Determination of Multiple Elements in Foods) [25], GB 5009.4-2016 (National Food Safety Standard—Determination of Ash in Foods) [26], and GB/T 5009.10-2003 (Determination of Crude Fiber in Plant-based Foods) [27]. Total antioxidant capacity was measured using the FRAP method as described by Sun et al. [28]. The original data obtained by this method were expressed in μmol/g. For a more intuitive representation of the relative content of antioxidant active substances in the samples, the molar concentration was converted into mass percentage (%). The conversion was calculated as follows: total antioxidant capacity (%) = total amount of antioxidants per gram of sample (μmol/g) × molar mass of ferrous sulfate × 0.001 × 100.

### 2.6. Evaluation of the Nutritional Value of Fruiting Body

The amino acid scoring (AAS), chemical score (CS), essential amino acid index (EAAI), and membership function method used in this study followed the same procedures as described in our previous research [19].

### 2.7. Statistical Analysis

Statistical analysis of the experimental data was performed using SPSS (IBM SPSS statistics 27) and Microsoft Excel 2021 software. Analysis of variance (ANOVA) was employed to assess the presence of significant differences in yield and quality among the samples, with a significance level set at *p* < 0.05.

## 3. Results and Discussion

### 3.1. Physicochemical Properties Analysis of Ginger Straw

Edible fungi accumulate elements from cultivation substrates through biological processes and adsorption during growth [29]. To investigate whether ginger straw contains heavy metals or pesticide residues, measurements were taken for lignin, cellulose, hemicellulose, crude fat, crude protein, volatile oils, curcumin, gingerol, carbon content, nitrogen content, potassium ions (K^+^), ash content, pH, five common heavy metals (arsenic, cadmium, mercury, lead, and chromium), and 100 common pesticide residue indicators. The results were presented in Appendix A Table A3, the physicochemical properties of ginger straw were similar to those of wheat straw, corn straw, and other crop residues, suggesting its potential as a primary component in edible fungus cultivation substrates [30,31]. Furthermore, the concentrations of the five heavy metals in ginger straw complied with safety standards, and none of the 100 common pesticide residues were detected.

### 3.2. Optimization of Ginger Straw Substrate Formulations

Based on a comprehensive analysis of the growth of *P. geesteranus* and *H. erinaceus* on PDA medium, the following strains were selected for optimizing ginger straw substrate formulations: *P. geesteranus* strains X2, X3, X4, X5, and X6; *H. erinaceus* strains H4, H9, and H10 (results are detailed in the Supplementary Materials). Using conventional cottonseed hull substrate as the control, the effects of ginger straw incorporation ratios (10, 15, 20, 25, 30, 35, and 40%) on the mycelial growth rate and vigor of *P. geesteranus* were investigated. The results (Table 1) showed variations in mycelial growth rate and vigor among different *P. geesteranus* strains across substrates with varying ginger straw ratios. Strains X2, X3, X4, X5, and X6 all exhibited the fastest growth rate in the conventional cottonseed hull substrate. X2 exhibited the slowest growth (5.06 mm d^−1^), which was observed at ginger straw ratios of 35% and 40%. Strains X3 (4.01 mm d^−1^), X4, X5, and X6 exhibited the slowest mycelial growth at a 40% ginger straw ratio. Furthermore, X3 displayed favorable mycelial characteristics, featuring dense and snow-white mycelium.

The growth rates and mycelial vigor of different *H. erinaceus* strains on media with varying ginger straw incorporation ratios are presented in Table 2. Strains H4, H9, and H10 exhibited the fastest growth rates in the conventional cottonseed hull substrate. For strains H4 and H9, the slowest growth rates were observed at a ginger straw incorporation ratio of 35%, whereas strain H10 showed the slowest growth at a 40% ginger straw ratio. Additionally, strain H10 displayed variations in mycelial color, density, and edge regularity across the different substrate formulations.

Based on the analysis of growth rate and mycelial vigor of different strains on ginger straw substrates, the following optimized formulations were determined. For *P. geesteranus* strains, the optimal ginger straw incorporation ratios were X2 and X4 at 25%, X3 at 30%, X5 at 15%, and X6 at 20%. *H. erinaceus* strains were cultivated on a 15% ginger straw substrate. Fruiting bodies are denoted as “strain code (ginger straw ratio),” e.g., X1(25) for 25% ginger straw substrate and X1(0) for conventional cottonseed hull substrate.

### 3.3. Effects of Ginger Straw on Fruiting Body Traits of Pleurotus geesteranus

#### 3.3.1. Effects of Commercial Traits and Biological Efficiency of Fruiting Bodies

**Figure 1:**
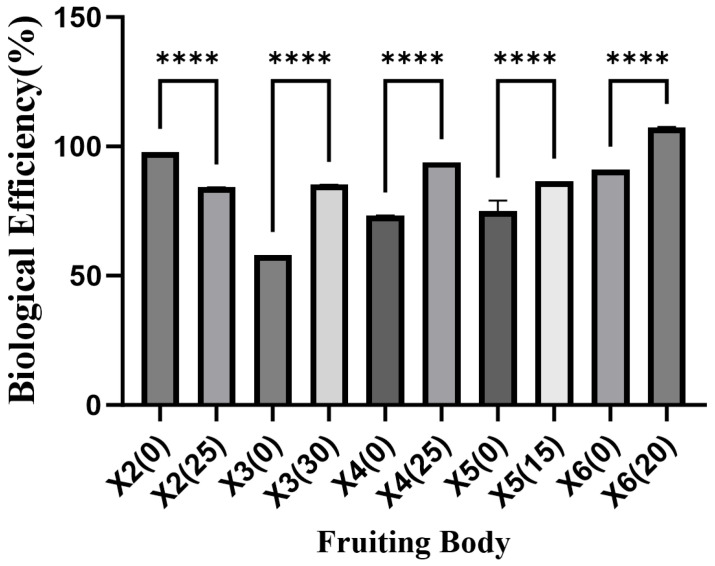
Biological Efficiency in the fruiting bodies of *Pleurotus geesteranus* (%), **** indicates highly significant difference (*p* < 0.0001).

Cultivation experiments demonstrated that the BE of *P. geesteranus* strains X3, X4, X5, and X6 grown on ginger straw substrate was significantly higher than that of those cultivated on cottonseed hull substrate, with increases of 27.1%, 20.71%, 9.08%, and 16.46%, respectively. In contrast, the BE of strain X2 grown on ginger straw substrate was significantly lower than that on cottonseed hull substrate, showing a reduction of 13.82% (Figure 1).

Next, we measured the commercial traits of *P. geesteranus*; the results were presented in Table 3. The ginger straw substrate influenced the stipe length, cap diameter, cap thickness, and moisture content of the fruiting bodies. For strains X3, X4, X5, and X6 cultivated on ginger straw substrate, the stipe length was slightly greater compared to those grown on cottonseed hull substrate. In contrast, strain X2 grown on ginger straw substrate exhibited a significantly shorter stipe length, with a reduction of 5.23%. The cap diameter of strain X6 cultivated on ginger straw substrate was significantly higher than that of the cottonseed hull-grown counterpart, showing an increase of 7.87%. For strains X3, X4, and X5, the cap diameter of ginger straw-grown fruiting bodies was larger than that from the cottonseed hull substrate, though the differences were not statistically significant. However, strain X2 showed a significantly smaller cap diameter when grown on ginger straw substrate, with a reduction of 8.65%. Ginger straw substrate significantly increased the cap thickness of strains X3 and X6, while no notable differences were observed for strains X2, X4, and X5. The moisture content of strain X2 fruiting bodies grown on ginger straw substrate was significantly higher than that of the cottonseed hull-grown ones, with an increase of 3.43%. In contrast, no significant differences in moisture content were observed between the two substrates for strains X3, X4, X5, and X6. When bamboo chips were used to replace 27% of cottonseed hulls for *P. geesteranus* cultivation, Chen et al. [32] reported a cap thickness of only 1.8–2.2 mm, which was significantly lower than that obtained using the ginger straw substrate in the present study. Using 15% *Pennisetum giganteum* to substitute sawdust, Lu et al. [33] achieved a relatively high yield of *P. geesteranus*, but the coefficient of variation for cap diameter was high, resulting in uneven morphological characteristics. In contrast, the ginger straw substrate produced *P. geesteranus* with no obvious deformities and a more uniform cap diameter. Regarding moisture content, the fruiting bodies cultivated on ginger straw substrate showed a value similar to the 88.06% reported by M Bonatti [34] for *Pleurotus ostreatus* grown on rice straw, falling within the high-quality standard range for edible fungi (85–92%). This indicates that ginger straw substrate effectively maintains water balance in fruiting bodies and improves commercial quality.

#### 3.3.2. Effects of the Nutritional Quality of Fruiting Bodies of *Pleurotus geesteranus*

##### Effects of Crude Protein, Crude Fat, Total Sugars, Reducing Sugars, Crude Fiber, and Ash Content

Crude protein, crude fat, total sugars, reducing sugars, crude fiber, and ash content serve as core nutritional indicators of edible fungus fruiting bodies [35,36]. In this study, fruiting bodies of *P. geesteranus* and *H. erinaceus*, cultivated on conventional cottonseed hull substrate as controls, were systematically investigated for the effects of ginger straw substrate on the contents of these six nutritional indicators in the fruiting bodies of both fungus species.

**Figure 2:**
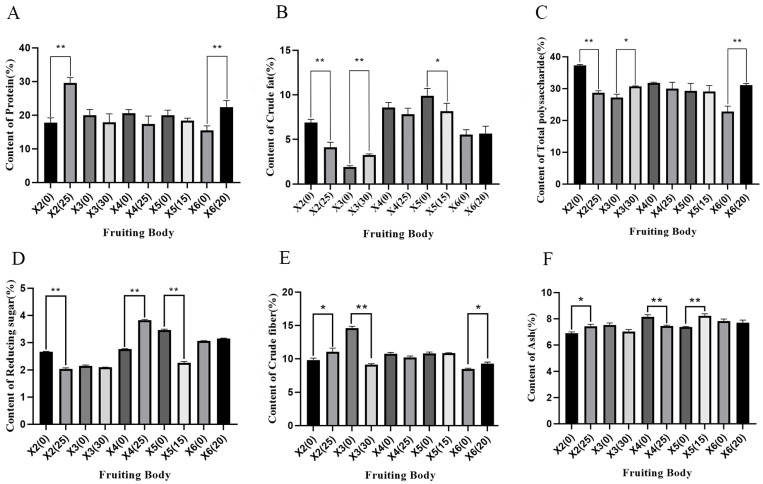
Contents of six nutrients in the fruiting bodies of *Pleurotus geesteranus* (%). Figure (**A**): Crude protein content of fruiting body (%). Figure (**B**): Crude fat content of fruiting body (%). Figure (**C**): Polysaccharide content of fruiting body (%). Figure (**D**): Reducing sugar content of fruiting body (%). Figure (**E**): Crude fiber content of fruiting body (%). Figure (**F**): Ash content of fruiting body (%). * indicated significant difference (*p* < 0.05), ** indicates extremely significant difference (*p* < 0.01).

As shown in Figure 2, compared with the cottonseed hull substrate, ginger straw influenced the crude protein content in the fruiting bodies of *P. geesteranus*. The crude protein content in fruiting bodies of strains X2 and X6 cultivated on ginger straw substrate was significantly higher than that in those grown on cottonseed hull substrate, with an increase of 11.79% and 6.92%, respectively. Although the crude protein content in fruiting bodies of strains X3, X4, and X5 grown on ginger straw substrate showed a decreasing trend compared to those on cottonseed hull substrate, the differences were not statistically significant. In the present study, the crude protein content of all tested fruiting bodies cultivated on ginger straw substrate was above 15%, which was significantly higher than that of *P. geesteranus* cultivated on corn straw substrate by Zhang [37]. This indicates that ginger straw is more conducive to improving protein accumulation and the nutritional value of *Pleurotus geesteranus*.

Among the five strains cultivated on ginger straw substrate, strain X3 exhibited a 1.41% increase in crude fat content, while strains X2 and X5 showed significant reductions of 2.8% and 1.8%, respectively. In contrast, the crude fat content in fruiting bodies of strains X4 and X6 was similar to that of those cultivated on cottonseed hull substrate. Ginger straw also influenced the total sugar content in the fruiting bodies of *P. geesteranus*. The total sugar content in ginger straw-cultivated fruiting bodies of strains X3 and X6 was significantly higher than that in cottonseed hull-cultivated ones, with an increase of 3.53% and 8.3%, respectively. In contrast, strain X2 showed a significantly lower total sugar content when cultivated on ginger straw substrate, with a reduction of 8.7%. For strains X4 and X5, the total sugar content in ginger straw-cultivated fruiting bodies was lower than that in cottonseed hull-cultivated ones, though the differences were not statistically significant. The total sugar content of ginger straw-cultivated *P. geesteranus* in this study was lower than that reported by Wang et al. for ten *P. pulmonarius* strains [38] and also lower than the levels observed by Wang et al. in *P. pulmonarius* cultivated on various forest waste substrates [39].

Compared to the cottonseed hull substrate, strain X4 showed an increase of 1.07% in reducing sugar content when cultivated on ginger straw substrate. In contrast, strains X2, X3, and X5 exhibited lower reducing sugar content in ginger straw-cultivated fruiting bodies compared to cottonseed hull-cultivated ones, while strain X6 showed higher reducing sugar content on ginger straw substrate. However, none of these differences were statistically significant. Ginger straw substrate also affected the crude fiber content of *P. geesteranus*. The crude fiber content in ginger straw-cultivated fruiting bodies of strains X2 and X6 was significantly higher than that in cottonseed hull-cultivated ones, with increases of 1.23% and 0.8%, respectively. In contrast, strains X3, X4, and X5 exhibited lower crude fiber content in ginger straw-cultivated fruiting bodies compared to cottonseed hull-cultivated ones. The crude fiber content of *P. geesteranus* cultivated on mulberry branch chips and Melia azedarach sawdust substrates was 8.33–8.48%, which was similar to that of *P. geesteranus* strains cultivated on ginger straw substrate in the present experiment [40]. In addition, compared with the crude fiber content of *P. geesteranus* cultivated on poplar sawdust substrate by Wang et al., the effect of ginger straw substrate on crude fiber content varied with strains, and the increase range of some strains (e.g., 1.23% for strain X2) was similar to the regulatory effect of forest waste substrates in that study [39]. The impact of ginger straw on the ash content of fruiting bodies was minimal for strains X2, X4, and X5, with variations all within 1%.

##### Effects of Ginger Straw on Amino Acid Content and Nutritional Value of *Pleurotus geesteranus* Fruiting Bodies

**Figure 3:**
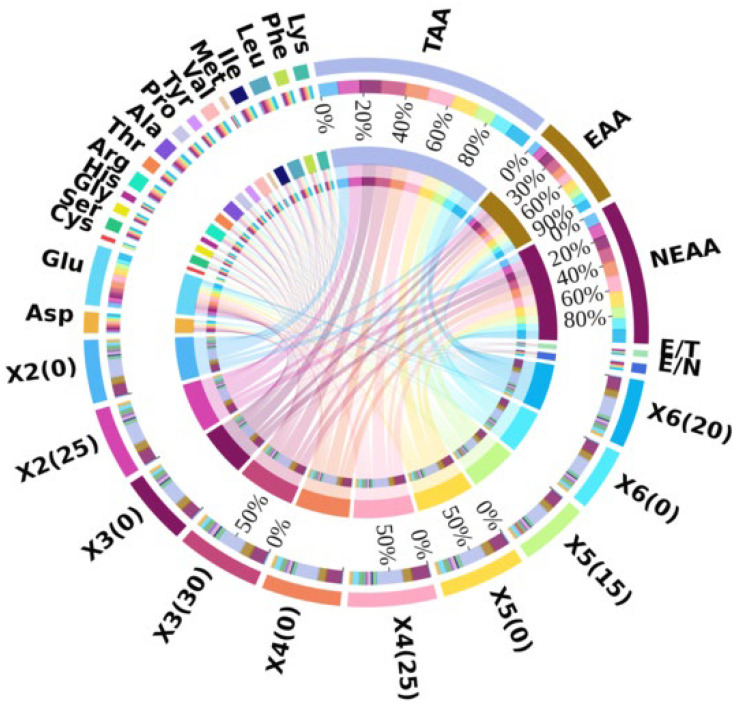
Circos analysis of hydrolyzed amino acid content of *Pleurotus geesteranus.* Note: total amino acid content of the fruiting body (TAA), essential amino acids (EAA), and non-essential amino acids (NEAA).

Ginger straw influenced the hydrolyzed amino acid content in *P. geesteranus* fruiting bodies (Figure 3). Compared to the cottonseed hull substrate, ginger straw cultivation increased the hydrolyzed amino acid content in strains X2, X3, and X4 by 2.28 mg g^−1^, 3.41 mg g^−1^, and 2.36 mg g^−1^, respectively. Strain X6 also showed higher hydrolyzed amino acid content with ginger straw, though the difference was not statistically significant. In contrast, strain X5 exhibited a significant decrease of 4.36 mg g^−1^ in hydrolyzed amino acid content when cultivated on ginger straw substrate. All tested fruiting bodies contained histidine and the other seven essential amino acids (EAAs) except tryptophan. Strains X2, X3, X4, and X6 demonstrated higher EAA content under ginger straw cultivation, with increases of 0.86 mg g^−1^, 1.59 mg g^−1^, 1.10 mg g^−1^, and 0.38 mg g^−1^, respectively, compared to cottonseed hull cultivation. However, strain X5 showed significantly lower EAA content when grown on ginger straw substrate.

The E/T (essential amino acids to total amino acids) ratios of all *P. geesteranus* fruiting bodies in this study were approximately 40%, while the E/N (essential amino acids to non-essential amino acids) ratios were around 60%, both meeting the ideal protein pattern recommended by FAO/WHO [41]. Among the strains, X3 and X4 showed slightly higher E/T and E/N values under ginger straw cultivation, though the differences were not significant. Strain X2 exhibited identical E/T and E/N values under both substrates, while strains X5 and X6 had slightly lower E/T and E/N values under ginger straw cultivation, with no significant differences observed.

The results in Table 4 showed that the contents of threonine, valine, isoleucine, phenylalanine, and tyrosine in all fruiting bodies exceeded the recommended values of FAO/WHO [41]. However, the contents of methionine and cysteine in all fruiting bodies, the leucine content in X4(0), X4(25), and X5(15), the lysine content in X2(25), X4(0), X5(0), X6(0), and X6(20), as well as the total essential amino acid content in X4(0), were lower than the FAO/WHO recommended values [42]. The total essential amino acids of all tested strains ranged from 34.98% to 41.86% of protein, which was lower than that of whole egg protein (49.7%). Among individual amino acids, Leu and Phe+Tyr in most samples were close to the levels in egg protein, while Met+Cys and Lys were relatively lower than the egg protein reference. Although the essential amino acid composition of the fruiting bodies was slightly lower than that of egg protein, the composition was complete and balanced, indicating that these strains still provide a valuable protein source complementary to daily diets [43]. Amino acid composition in ginger straw-cultivated edible fungus fruiting bodies was evaluated using amino acid scoring (AAS). The results (Table 5) indicated that methionine and cysteine were the first limiting amino acids in *P. geesteranus* fruiting bodies. Compared to cottonseed hull cultivation, the AAS scores of strains X3 and X4 grown on ginger straw substrate increased by 0.02 and 0.04, respectively, though the differences were not statistically significant. In contrast, strains X2, X5, and X6 showed decreases in AAS scores of 0.01, 0.03, and 0.03, respectively. Notably, all AAS scores of ginger straw-cultivated fruiting bodies were above 1, indicating higher amino acid value and better digestibility [44].

Chemical score (CS) evaluation of the fruiting bodies (Table 6) further confirmed that methionine and cysteine remained the first limiting amino acids, with CSs ranging from 0.29 to 0.35. Compared to cottonseed hull cultivation, ginger straw substrate increased the CSs of strains X3, X4, and X6 by 0.02, 0.03, and 0.01, respectively, though the differences were not statistically significant. In contrast, strain X5 showed a slight decrease of 0.01 in CS, while strain X2 exhibited no change in CS between the two substrates. Regarding the essential amino acid index (EAAI), ginger straw-cultivated fruiting bodies of strains X3 and X4 showed an increase of 0.01 compared to those grown on cottonseed hull substrate, while strain X6 showed a decrease of 0.02. However, these differences were not statistically significant. No significant differences in EAAI were observed between ginger straw and cottonseed hull substrates for strains X2 and X5. Importantly, all ginger straw-cultivated fruiting bodies exhibited EAAI values above 0.95, indicating their potential as a high-quality protein source [45].

**Figure 4:**
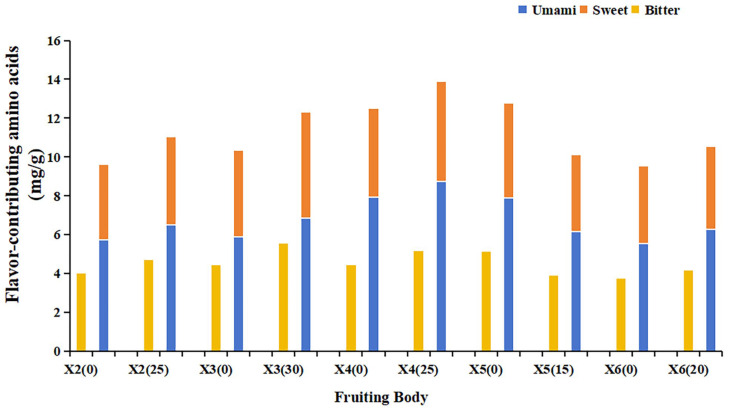
Content of flavor-contributing amino acids of *Pleurotus geesteranus* on the cottonseed hull substrate and ginger straw substrate (mg g^−1^).

Analysis of the content and proportion of umami, sweet, and bitter amino acids in *P. geesteranus* fruiting bodies is revealed in Figure 4. Ginger straw substrate significantly increased the content of umami amino acids in strains X2, X3, X4, and X6, as well as the content of sweet amino acids in strains X2, X3, and X4. Concurrently, it significantly reduced the content of bitter amino acids in strain X5. These findings indicate that ginger straw-cultivated *P. geesteranus* fruiting bodies possess favorable flavor and taste characteristics [46].

**Figure 5:**
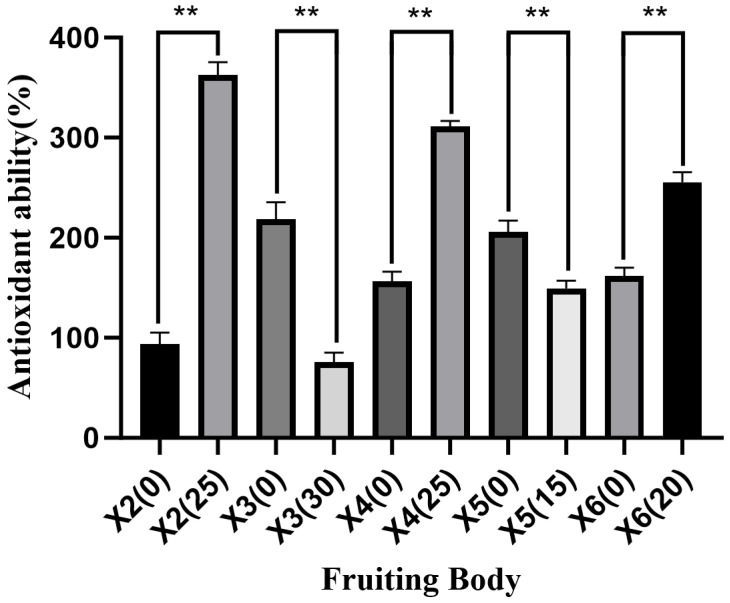
Antioxidant ability of fruiting body (%) Note: ** indicated extremely significant difference (*p* < 0.01).

The addition of ginger straw to the cultivation substrate enhanced the total antioxidant capacity of *P. geesteranus* fruiting bodies (Figure 5). Specifically, the total antioxidant capacity of ginger straw-cultivated fruiting bodies from strains X2, X4, and X6 was significantly higher than that of cottonseed hull-cultivated ones, with increases of 268.84%, 154.83%, and 93.86%, respectively. In contrast, strains X3 and X5 exhibited significantly lower total antioxidant capacity when cultivated on ginger straw substrate, with reductions of 143.17% and 56.21%, respectively. In previous studies, the antioxidant capacity of *Pleurotus eryngii* fruiting bodies cultivated on a new substrate mixed with coffee grounds and thyme was also significantly improved [47], which was similar to the findings of *P. geesteranus* in the present study. These results indirectly demonstrate that ginger straw, as a component of cultivation substrate, exerts a positive effect on the nutritional value of *P. geesteranus* fruiting bodies.

##### Effects of Ginger Straw Substrate on Trace Element Content in *Pleurotus geesteranus* Fruiting Bodies

**Figure 6:**
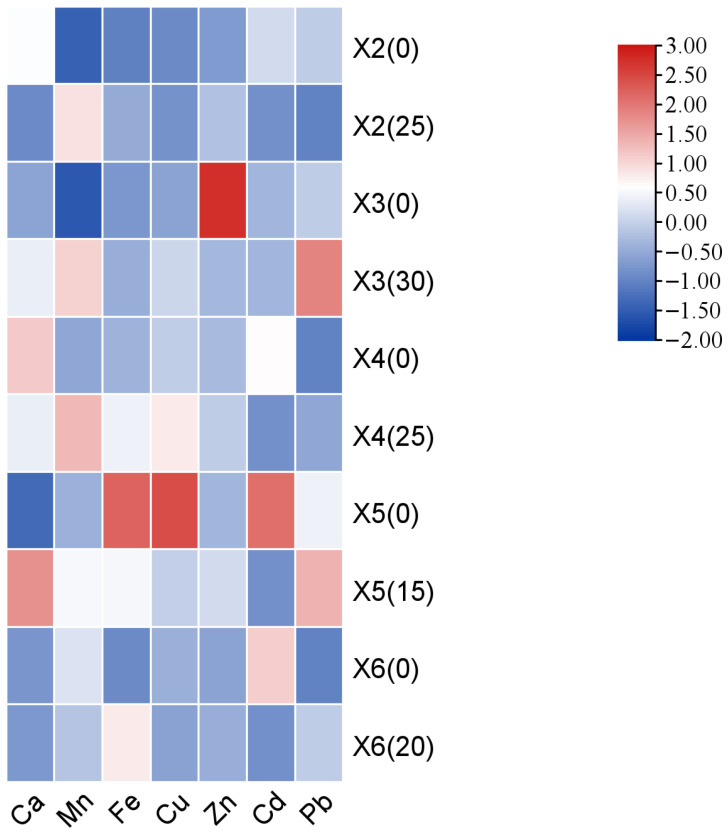
Heat map analysis of the nutritional components of *Pleurotus geesteranus* on the cottonseed hull substrate and GSS.

Figure 6 showed that the fruiting bodies of all strains were rich in Ca, Mn, Fe, Cu, and Zn. Among these, Ca was generally the most abundant element, with concentrations ranging from 205.42 mg kg^−1^ to 409.92 mg kg^−1^. Compared to cottonseed hull cultivation, ginger straw substrate significantly increased the Ca content in strains X3 and X5 by 64.61 mg kg^−1^ and 204.50 mg kg^−1^, respectively. Strain X6 also exhibited higher Ca content with ginger straw, though the difference was not statistically significant. In contrast, strains X2 and X4 showed significantly lower Ca content under ginger straw cultivation, with reductions of 98.89 mg kg^−1^ and 50.32 mg kg^−1^, respectively. Additionally, ginger straw substrate increased the Mn content in strains X2, X3, X4, and X5; the Fe content in strains X2, X3, X4, and X6; the Cu content in strains X2, X3, and X4; and the Zn content in strains X2, X4, X5, and X6.

Cd and Pb are common heavy metal elements harmful to human health [48]. In the ginger straw-cultivated fruiting bodies, Cd content was significantly lower in strains X2, X4, X5, and X6 compared to cottonseed hull cultivation, with reductions of 0.02 mg kg^−1^, 0.03 mg kg^−1^, 0.06 mg kg^−1^, and 0.04 mg kg^−1^, respectively. Pb content varied significantly among the different fruiting bodies. Strain X2 showed lower Pb content under ginger straw cultivation, though the difference was not significant. In contrast, strains X3, X4, X5, and X6 exhibited higher Pb content with ginger straw. Among these, strain X3 showed a significant increase of 0.04 mg kg^−1^, while no significant differences in Pb content were observed between the two substrates for strains X4, X5, and X6.

### 3.4. Effects of Ginger Straw on Fruiting Body Traits of Hericium erinaceus

#### 3.4.1. Effects of Commercial Traits and Biological Efficiency of Fruiting Bodies

**Figure 7:**
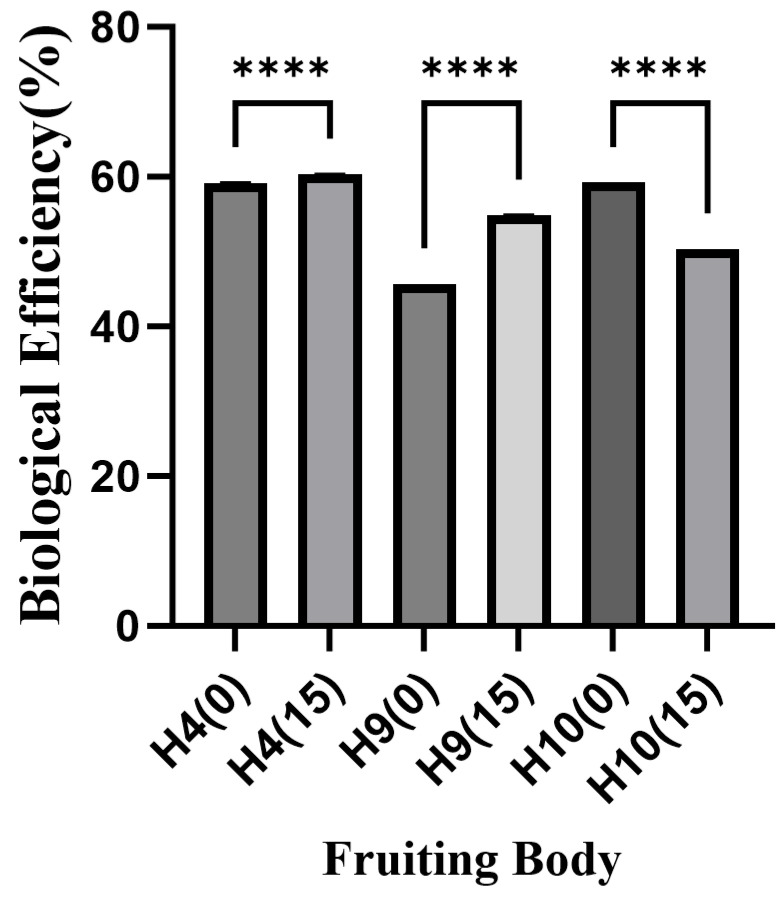
Biological efficiency in the fruiting bodies of *Hericium erinaceus* (%), **** indicates highly significant difference (*p* < 0.0001).

Using conventional cottonseed hull substrate as the control, the BE of *H. erinaceus* fruiting bodies cultivated on ginger straw substrate was analyzed. As shown in Figure 7, ginger straw significantly improved the BE of strain H9 by 9.16%, while a slight increase of 1.23% was observed for strain H4. In contrast, the biological efficiency of strain H10 decreased significantly by 8.87% under ginger straw cultivation. Compared with the BE of *H. erinaceus* cultivated on sawdust substrate replaced by 20–83% hemp straw, the BE in the present study was relatively lower [16]. However, the addition of ginger straw resulted in a higher BE of *H. erinaceus* than that of the control group. The changes in commercial traits of *H. erinaceus* followed a similar trend to the BE results (Table 7). Ginger straw substrate increased the cap diameter of strains H4 and H9 by 5.49 mm and 3.61 mm, respectively, with the difference for strain H9 being statistically significant. However, the cap diameter of strain H10 cultivated on ginger straw substrate was significantly smaller than that of the cottonseed hull-cultivated counterpart, showing a reduction of 15.29 mm. No significant differences in moisture content were observed between the two substrates for any of the tested *H. erinaceus* strains.

#### 3.4.2. Effects of the Nutritional Quality of Fruiting Bodies of *Hericium erinaceus*

##### Effects of Crude Protein, Crude Fat, Total Sugars, Reducing Sugars, Crude Fiber, and Ash Content

**Figure 8:**
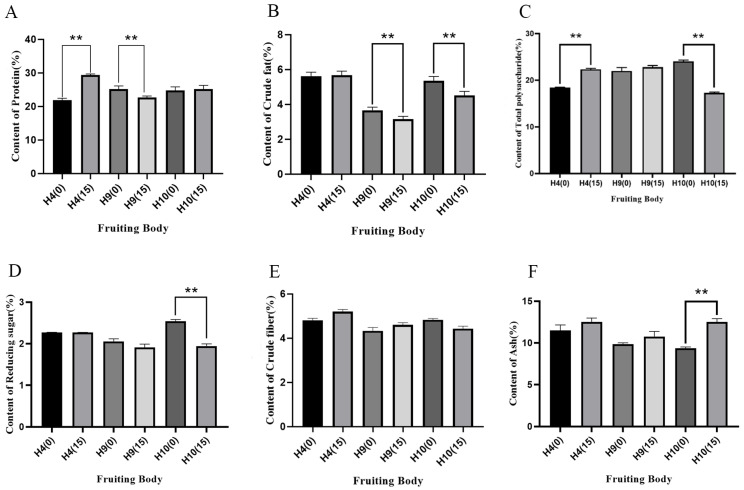
Contents of six nutrients in the fruiting bodies of *H. erinaceus* (%). Figure (**A**): Crude protein content of fruiting body (%). Figure (**B**): Crude fat content of fruiting body (%). Figure (**C**): Polysaccharide content of fruiting body (%). Figure (**D**): Reducing sugar content of fruiting body (%). Figure (**E**): Crude fiber content of fruiting body (%). Figure (**F**): Ash content of fruiting body (%). ** indicates extremely significant difference (*p* < 0.01).

Ginger straw exerted a highly significant effect on the crude protein content in the fruiting bodies of H4 and H9 strains. The crude protein content in strain H4 increased significantly by 7.51%, while in strain H9, it decreased significantly by 2.47%. In contrast, strain H10 showed a slight increase of 0.38% in crude protein content, though the difference was not statistically significant (Figure 8). These findings suggest that ginger straw can modestly enhance the crude protein content in *H. erinaceus* fruiting bodies, and the crude protein level of *H. erinaceus* strains in this experiment was much higher than that of *H. erinaceus* cultivated with hemp straw by Zhang et al. [16].

The addition of ginger straw also influenced the crude fat content in *H. erinaceus* fruiting bodies. The crude fat content in ginger straw-cultivated fruiting bodies of strains H9 and H10 was significantly lower than that in cottonseed hull-cultivated ones, with reductions of 0.51% and 0.83%, respectively. In contrast, strain H4 showed a slight increase of 0.04% in crude fat content under ginger straw cultivation, though the difference was not statistically significant. Compared to the reasonable range of crude fat content in *H. erinaceus* reported by Friedman, the crude fat content in some ginger straw-cultivated strains in this study was slightly higher [49]. This may be attributed to the utilization of small amounts of fat precursors (e.g., fatty acid methyl esters) present in ginger straw by the fungi [49]. Additionally, as noted by Tasgaonkar et al., *H. erinaceus* fruiting bodies contain fatty acid components such as palmitic acid, which might be further induced for synthesis in ginger straw substrate, leading to a slight increase in crude fat content in some strains [50].

Ginger straw substrate had varying effects on the total sugar content in the fruiting bodies of *H. erinaceus*. The total sugar content in strain H4 increased significantly by 3.9%, while in strain H10 it decreased significantly by 6.77%. Strain H9 showed an increase of 0.85%, though this difference was not statistically significant. The effect on reducing sugar content in the fruiting bodies was relatively minor. Strain H10 exhibited a significant reduction of 0.6%, whereas strains H4 and H9 showed decreases of 0.01% and 0.15%, respectively. The results for crude fiber content showed that the crude fiber content in ginger straw-cultivated fruiting bodies of strains H4 and H9 was higher than that in cottonseed hull-cultivated ones, with increases of 0.4% and 0.24%, respectively. In contrast, strain H10 exhibited a reduction of 0.4% in crude fiber content under ginger straw cultivation. However, the differences between the two substrates were not statistically significant for any of the three strains. Friedman reported that the reasonable range for crude fiber content in *H. erinaceus* was 2.5–8.5%. In this study, the crude fiber content of all strains fell within this range and showed no abnormal fluctuations, indicating that ginger straw substrate did not compromise the dietary fiber supply characteristics of *H. erinaceus* [49].

Meanwhile, ginger straw increased the ash content in the fruiting bodies of *H. erinaceus*. A highly significant increase of 3.14% was observed in strain H10, while strains H4 and H9 showed moderate increases of 1.03% and 0.89%, respectively, though these differences were not statistically significant. Moreover, the ash content of *H. erinaceus* strains in this experiment was superior to that of *H. erinaceus* cultivated with hemp straw by Zhang et al. [16].

##### Effects of Ginger Straw on Amino Acid Content and Nutritional Value of *Hericium erinaceus* Fruiting Bodies

**Figure 9:**
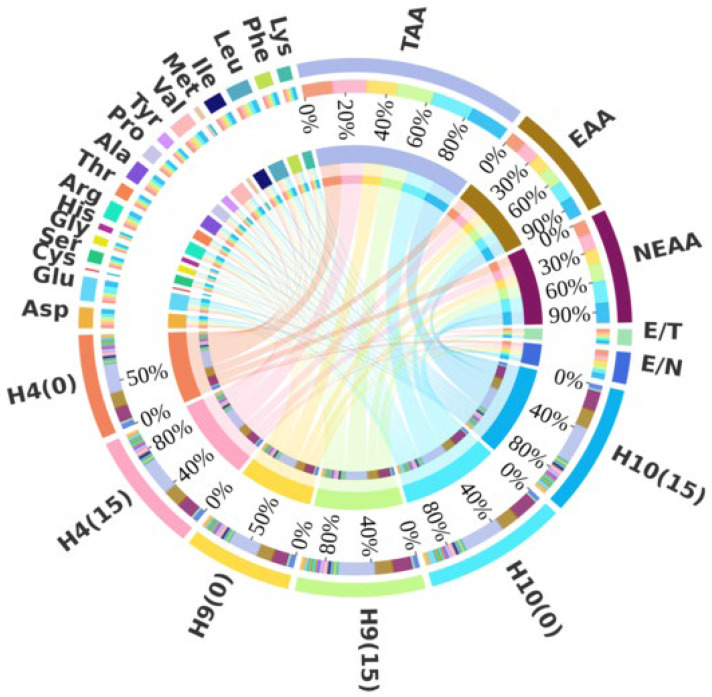
Circos analysis of hydrolyzed amino acid contentof *Hericium erinaceus.* Note: total amino acid content of the fruiting body (TAA), essential amino acids (EAA), and non-essential amino acids (NEAA).

To evaluate the protein quality of *H. erinaceus* fruiting bodies cultivated on ginger straw substrate, the hydrolyzed amino acid content in different fruiting bodies was determined. As shown in Figure 9, the total hydrolyzed amino acid content in ginger straw-cultivated fruiting bodies of strains H4 and H9 was significantly higher than that in cottonseed hull-cultivated ones, with increases of 0.99 mg g^−1^ and 1.41 mg g^−1^, respectively. In contrast, strain H10 exhibited a significant reduction of 0.64 mg g^−1^ in hydrolyzed amino acid content under ginger straw cultivation. The EAA content in ginger straw-cultivated *H. erinaceus* fruiting bodies was generally higher than that in cottonseed hull-cultivated ones. Specifically, strains H4 and H9 showed significant increases of 0.47 mg g^−1^ and 0.56 mg g^−1^, respectively, while strain H10 exhibited a slight increase of 0.04 mg g^−1^, though the difference was not statistically significant. In this study, the E/T ratios of all *H. erinaceus* fruiting bodies exceeded 40%, and the E/N ratios exceeded 60%, meeting the ideal protein pattern recommended by FAO/WHO [41]. Notably, strain H10 showed significantly higher E/T and E/N values under ginger straw cultivation compared to cottonseed hull cultivation. In contrast, strains H4 and H9 exhibited slightly lower E/T and E/N values under ginger straw cultivation, though the differences were not statistically significant.

The analysis of essential amino acid composition and nutritional value of proteins in *H. erinaceus* fruiting bodies revealed that the contents of threonine, valine, isoleucine, leucine, phenylalanine, tyrosine, and total essential amino acids in all fruiting bodies exceeded the recommended values of FAO/WHO [41]. In contrast, the contents of methionine and cysteine in all fruiting bodies, as well as the lysine content in H4(0), H4(15), H9(0), and H10(0), were lower than the FAO/WHO recommended values (Table 8) [41]. The TEAA in fruiting bodies of the tested strains ranged from 45.42% to 52.56% of crude protein. Most strains contained higher or similar TEAA compared with egg protein (49.7%), indicating abundant essential amino acids. Contents of Val, Ile, Leu, and Phe+Tyr in all strains were higher than those in egg protein, showing a balanced amino acid profile. Only Met+Cys and Lys were lower than egg protein and were recognized as the main limiting amino acids. Overall, the tested strains exhibited a complete and balanced essential amino acid profile with high nutritional value and could be used as high-quality protein resources from edible fungi [43]. The amino acid composition in the fruiting bodies of edible fungi cultivated from ginger straw was determined by AAS. The results showed that methionine and cystine were the first limiting amino acids in the fruiting bodies of *H. erinaceus* (Table 9). The AAS score of the fruiting body of ginger straw with strain H10 was higher than that of cottonseed shell, with an increase of 0.12, and the difference was significant. The AAS scores of the fruiting body of ginger straw with strains H4 and H9 were lower than those of cottonseed shell, with a decrease of 0.03 and 0.07, respectively, and the difference was not significant. The AAS score values of all ginger straw fruiting bodies of *H. erinaceus* were higher than 1, which indicates that the amino acid value of the ginger straw fruiting bodies obtained in this study is relatively high and conducive to digestion and absorption. Chemical score (CS) evaluation of the fruiting bodies (Table 10) confirmed that methionine and cysteine remained the first limiting amino acids, consistent with the AAS results. The CSs ranged from 0.27 to 0.50. Strain H10 exhibited a significant increase of 0.08 in CS under ginger straw cultivation compared to cottonseed hull cultivation. In contrast, strains H4 and H9 showed decreases of 0.02 and 0.05 in CSs, respectively, though these differences were not statistically significant. The EAAI value of strain H10 cultivated on ginger straw substrate was higher than that of the cottonseed hull substrate, with an increase of 0.02, and the difference was significant. In contrast, the EAAI value of strain H9 cultivated on ginger straw substrate was lower than that of the cottonseed hull substrate, with a decrease of 0.01, though the difference was not statistically significant. Strain H4 showed no difference in EAAI value between the ginger straw and cottonseed hull substrates. Furthermore, the EAAI values of all ginger straw-cultivated fruiting bodies in this study were above 0.95, indicating their potential as a high-quality protein source.

**Figure 10:**
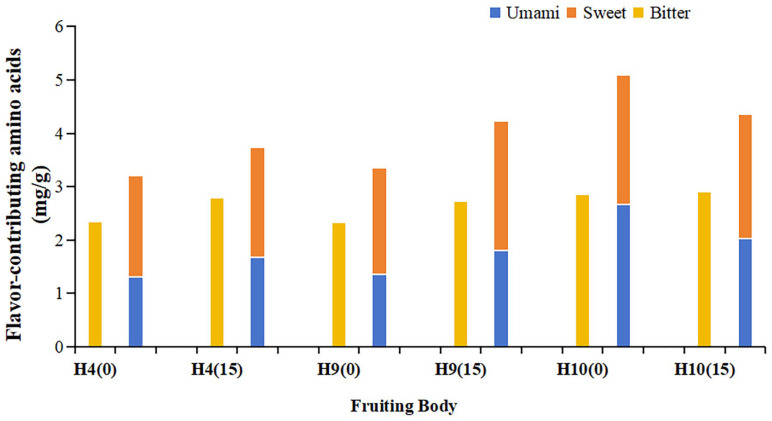
Content of flavor-contributing amino acids of *Hericium erinaceus* on the cottonseed hull substrate and ginger straw substrate (mg g^−1^).

The flavor amino acids in *H. erinaceus* fruiting bodies were analyzed, and the results are shown in Figure 10. Ginger straw significantly increased the contents of umami, sweet, and bitter amino acids in strains H4 and H9, while significantly reducing the umami amino acid content in strain H10. Although bitter amino acids accounted for a relatively high proportion in the ginger straw-cultivated fruiting bodies obtained in this study, their total content was lower than the combined content of umami and sweet amino acids. This indirectly indicates that ginger straw-cultivated *H. erinaceus* fruiting bodies possess favorable flavor characteristics.

**Figure 11:**
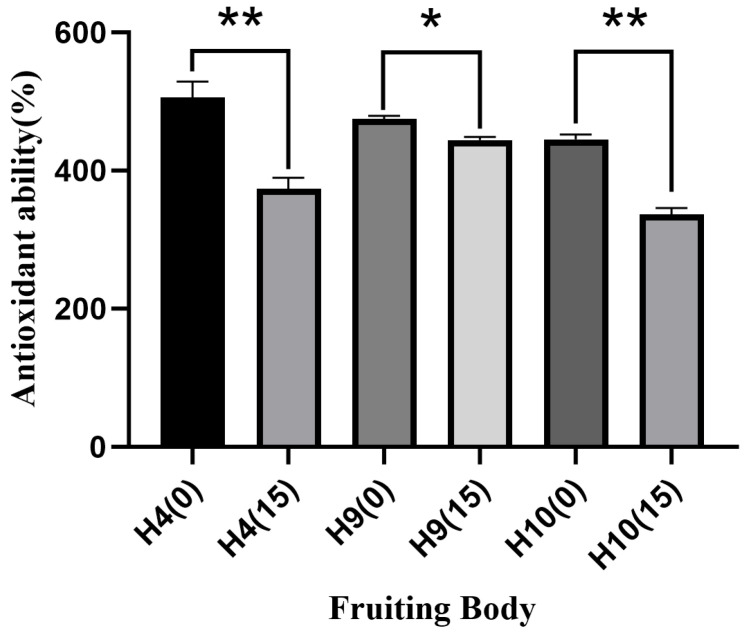
Antioxidant ability of fruiting body of *Hericum erinaceus* (%) Note: * indicates significant difference (*p* < 0.05), ** indicates extremely significant difference (*p* < 0.01).

However, the ginger straw substrate reduced the total antioxidant capacity of *H. erinaceus* fruiting bodies (Figure 11). The total antioxidant capacity of strains H4 and H10 decreased significantly by 132.34% and 108.71%, respectively, while strain H9 showed a significant reduction of 30.22%.

**Figure 12:**
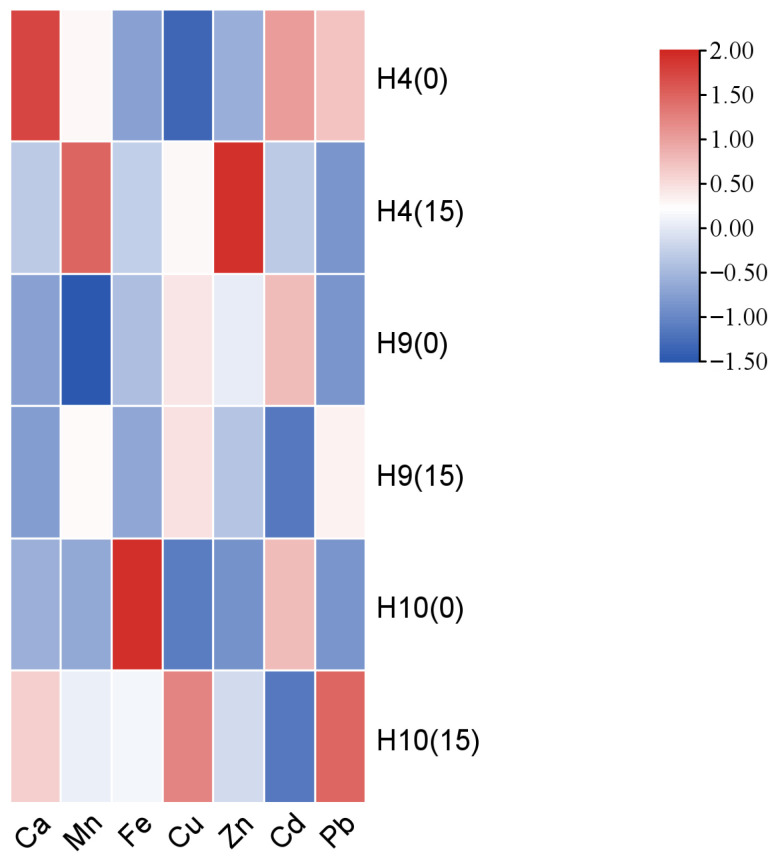
Heat map analysis of the nutritional components of *Hericium erinaceus* on the cottonseed hull substrate and GSS.

To investigate the distribution and accumulation of minerals during the production of *H. erinaceus*, the mineral composition in the fruiting bodies was analyzed, and the results are shown in Figure 12. The fruiting bodies were rich in Ca, Mn, Fe, Cu, and Zn. Among these, Ca content was relatively high, ranging from 137.39 mg kg^−1^ to 238.43 mg kg^−1^. Notably, the Ca content in the ginger straw-cultivated fruiting bodies of strain H10 was significantly higher than that in the cottonseed hull-cultivated ones, with an increase of 43.07 mg kg^−1^. Furthermore, the ginger straw substrate significantly increased the Mn, Fe, Cu, and Zn contents in strain H4. It also significantly enhanced the Mn content in strain H9 and the Mn, Cu, and Zn contents in strain H10. Additionally, the ginger straw substrate significantly reduced the Cd content in all *H. erinaceus* fruiting bodies and significantly lowered the Pb content in strain H4. Importantly, the Cd and Pb contents in all ginger straw-cultivated fruiting bodies complied with safety standards.

### 3.5. Comprehensive Quality Evaluation of Edible Fungi Using the Membership Function Method

The membership function method is a basic concept in fuzzy mathematics, and it often serves as the first step in solving practical problems using fuzzy mathematics [51]. This method is widely applied in the field of comprehensive plant evaluation and has been used for the identification and selection of various crop resources, such as seedless grapes, sorghum, corn, sugarcane, and rice [52,53,54]. However, its application in the development of new cultivation substrates for edible fungi is rarely reported.

Previously, Li et al. used the membership function comprehensive evaluation system to screen and identify new garden-waste-based cultivation substrates for *Stropharia rugosoannulata* and successfully invented a new formula for *Stropharia rugosoannulata* cultivation [55]. Based on the amino acid content of commercially available edible fungi, Wang et al. ranked them using the membership function comprehensive evaluation system and divided the comprehensive quality of commercially available edible fungi into three grades (Grade I, II, and III), providing a reference for citizens to select edible fungi as food [56].

Ginger straw had varying effects on the overall quality of *P. geesteranus* fruiting bodies (Table 11). The average membership function values for strains X2, X3, and X4 increased by 0.04, 0.02, and 0.01, respectively. The overall average membership function value for ginger straw-cultivated *P. geesteranus* fruiting bodies was 0.497, compared to 0.484 for cottonseed hull-cultivated ones. These results indicated that ginger straw substrate can enhance the comprehensive quality of *P. geesteranus* fruiting bodies.

Similarly, the overall quality of *H. erinaceus* was evaluated using the membership function method. The results (Table 12) showed that ginger straw substrate improved the comprehensive quality of strain H4, with its average membership function value increasing from 0.49 to 0.52, a gain of 0.03. However, the overall average membership function value for ginger straw-cultivated *H. erinaceus* fruiting bodies was slightly lower than that of cottonseed hull-cultivated ones, decreasing from 0.484 to 0.481, a reduction of only 0.003.

The findings of this study suggested that ginger straw substrate is suitable for cultivating *P. geesteranus* strains X2, X3, and X4, as well as *H. erinaceus* strain H4. Although ginger straw substrate did not significantly improve the comprehensive quality of *P. geesteranus* strains X5 and X6 or *H. erinaceus* strains H9 and H10, its use as a cultivation substrate holds research value from the perspective of waste utilization.

Based on the average membership function values and the above comprehensive evaluation results, the differences in growth and quality of the two edible fungi on ginger straw substrate are essentially due to their species-specific biological characteristics and genetic background differences, which are elaborated as follows.

*P. geesteranus* belongs to the *genus Pleurotus* (a typical white-rot fungus), whose core advantage is a highly active lignocellulolytic enzyme system, the basis for its efficient utilization of ginger straw [57]. The genome of *Pleurotus* fungi contains a large number of functional genes related to lignocellulose degradation, which can rapidly decompose cellulose and hemicellulose in ginger straw; meanwhile, its nutrient metabolism-related genes have high plasticity, enabling them to respond to high contents of crude protein, ash and other nutrients in ginger straw, improving fruiting body yield and quality—this is also the key reason why *P. geesteranus* strains X2, X3, and X6 perform optimally on 30% ginger straw substrate [58].

*H. erinaceus* differs significantly from *P. geesteranus*, with lower cellulase activity and higher substrate sensitivity, and its optimal ginger straw addition ratio is 15% (excess will inhibit growth) [59]. Genetically, it has a lower copy number and expression efficiency of lignocellulose degradation-related genes and weaker plasticity of nutrient metabolism genes, resulting in lower utilization efficiency of ginger straw nutrients than *P. geesteranus*. Thus, the increase in its BE (45.69–54.85%) is lower than that of *P. geesteranus* strain X3 (58.03–85.13%) [60].

Interspecific differences also exist in their nutrient and flavor regulation mechanisms: the expression of genes related to umami and sweet amino acid synthesis in *P. geesteranus* is more significantly induced by nitrogen sources, leading to better flavor improvement; both take methionine and cysteine as the first limiting amino acids (restricted by genetic conservation), but the sufficient nitrogen source in ginger straw can increase their overall amino acid content, making the amino acid score > 1 and essential amino acid index > 0.95, improving protein nutritional value [61,62].

## 4. Conclusions and Prospects

Crop straw has been widely adopted as a substrate in edible fungus cultivation. Extensive research confirms that common agricultural straws—such as corn straw, wheat straw, and rice straw—are suitable alternative substrates, demonstrating favorable performance in terms of mycelial growth rate, fruiting body yield, and quality [63,64,65,66,67]. This utilization model not only addresses the challenge of recycling straw resources but also alleviates cost pressures in the fungus industry caused by rising prices of traditional raw materials. The ginger straw used in this study was analyzed and found to contain levels of cellulose, hemicellulose, and lignin comparable to those of wheat straw and corn straw. Its crude protein and ash content were higher than those of conventional corn straw, wheat straw, and cotton straw, while its crude fat content was similar to that of these straws [30,68,69,70]. This study systematically demonstrated that ginger straw can serve as a high-quality novel substrate for cultivating *P. geesteranus* and *H. erinaceus*.

Specifically, strains X2, X3, and X6 of *P. geesteranus* and strains H9 and H10 of *H. erinaceus* cultivated on ginger straw substrate showed significantly higher average membership function values than those grown on traditional cottonseed hull substrate, indicating an improvement in overall quality. Compared with the traditional cottonseed hull substrate, the two tested edible fungi exhibited higher BE on the ginger straw-based substrate. For instance, the BE of strain X3 was 58.03% in the traditional cottonseed hull substrate, while it significantly increased to 85.13% in the composite substrate supplemented with 30% ginger straw; the BE of H9 was 45.69% in the traditional cottonseed hull substrate, and it increased to 54.85% in the composite substrate supplemented with 15% ginger straw. These results further confirm that ginger straw, as an alternative substrate for edible fungi cultivation, has good technical feasibility and practical application effectiveness and can effectively improve the yield potential of edible fungi.

In terms of nutritional and flavor profiles, ginger straw substrate not only significantly increased the content of nutrients such as crude protein, reducing sugars, crude fiber, and total antioxidant capacity in certain strains of both fungus species but also enhanced the accumulation of minerals such as potassium, calcium, and magnesium. Moreover, by increasing the total content of umami and sweet amino acids while reducing bitter amino acids, the substrate positively influenced the flavor characteristics of the fungi. Additionally, the amino acid scores of both *P. geesteranus* and *H. erinaceus* exceeded 1, and chemical scoring identified methionine and cysteine as the first limiting amino acids for both species, with essential amino acid indices all above 0.95. These results underscore the significant advantage of ginger straw substrate in improving the protein nutritional value of edible fungi.

This study demonstrated that ginger straw can be used as a viable substrate component for the cultivation of *P. geesteranus* and *H. erinaceus.* Different substrate formulas significantly affected mycelial growth, fruiting body yield, and BE, and obvious interspecific differences were observed between the two fungi species. The optimal proportion of ginger straw varied with strain and species, which was closely related to their genetic characteristics, phenotypic plasticity, and substrate decomposition ability. These results provide a theoretical basis and practical reference for the resource utilization of ginger straw and the sustainable development of edible fungi cultivation [71,72,73].

In summary, this study showed that ginger straw, as a novel cultivation substrate, can effectively increase the yield, optimize the agronomic traits, and enhance the nutritional quality of *P. geesteranus* and *H. erinaceus*. The findings provided a viable pathway for the efficient resource utilization of ginger straw and established a solid theoretical foundation for the development of innovative substrates in edible fungus cultivation.

## Figures and Tables

**Table 1:** The in situ growth rate and growth of *Pleurotus geesteranus* strains in different proportions of ginger straw medium.

Strains	Supplemented Proportion of Ginger (%)	Growth Rate (mm d^−1^)	Color	Marginal Regularity	Density	Growth
X2	0	7.61 ± 0.52 ^a^	snow-white	relatively neat	dense	++
	10	7.18 ± 0.09 ^ab^	snow-white	relatively neat	dense	++
	15	6.81 ± 0.17 ^bc^	snow-white	relatively neat	dense	++
	20	6.65 ± 0.58 ^c^	snow-white	neat	dense	+++
	25	6.42 ± 0.09 ^c^	snow-white	neat	dense	+++
	30	5.68 ± 0.26 ^d^	snow-white	neat	relatively dense	++
	35	5.06 ± 0.28 ^d^	snow-white	relatively neat	dense	++
	40	5.06 ± 0.22 ^d^	white	relatively neat	relatively dense	+
X3	0	7.65 ± 0.48 ^a^	snow-white	neat	dense	+++
	10	7.18 ± 0.13 ^ab^	snow-white	neat	dense	+++
	15	6.96 ± 0.066 ^abc^	snow-white	relatively neat	dense	++
	20	7.08 ± 0.24 ^abc^	snow-white	relatively neat	dense	++
	25	6.29 ± 0.13 ^c^	snow-white	neat	dense	+++
	30	6.34 ± 0.11 ^bc^	snow-white	neat	dense	+++
	35	5.11 ± 0.07 ^d^	snow-white	relatively neat	dense	++
	40	4.01 ± 0.56 ^e^	snow-white	neat	relatively dense	+
X4	0	7.67 ± 0.62 ^a^	snow-white	neat	dense	+++
	10	7.33 ± 0.05 ^a^	snow-white	untidy	dense	++
	15	7.39 ± 0.07 ^a^	snow-white	neat	dense	+++
	20	7.22 ± 0.08 ^a^	snow-white	relatively neat	dense	++
	25	7.11 ± 0.42 ^a^	snow-white	neat	dense	+++
	30	6.12 ± 0.06 ^b^	snow-white	relatively neat	dense	++
	35	5.33 ± 0.09 ^c^	snow-white	relatively neat	dense	++
	40	4.32 ± 0.25 ^d^	snow-white	relatively neat	relatively dense	++
X5	0	8.01 ± 0.09 ^a^	snow-white	untidy	dense	++
	10	7.69 ± 0.22 ^b^	snow-white	untidy	dense	++
	15	7.33 ± 0.09 ^b^	snow-white	neat	dense	+++
	20	6.47 ± 0.06 ^c^	snow-white	relatively neat	dense	++
	25	6.49 ± 0.07 ^c^	snow-white	relatively neat	dense	++
	30	6.13 ± 0.16 ^d^	snow-white	relatively neat	dense	++
	35	5.98 ± 0.22 ^d^	snow-white	neat	relatively dense	++
	40	5.31 ± 0.28 ^e^	snow-white	relatively neat	relatively dense	+
X6	0	7.98 ± 0.47 ^a^	snow-white	neat	dense	+++
	10	7.25 ± 0.3 ^b^	snow-white	untidy	dense	++
	15	7.05 ± 0.1 ^bc^	snow-white	untidy	dense	++
	20	6.59 ± 0.08 ^cd^	snow-white	neat	dense	+++
	25	6.49 ± 0.03 ^d^	snow-white	relatively neat	dense	++
	30	6.37 ± 0.11 ^d^	snow-white	relatively neat	dense	++
	35	6.27 ± 0.17 ^d^	snow-white	neat	relatively dense	++
	40	4.69 ± 0.28 ^e^	snow-white	relatively neat	relatively dense	+

Note: “+”—poor growth; “++”—the growth is average; “+++”—the growth is better than average. Different lowercase letters indicate significant differences, *p* < 0.05.

**Table 2:** The in situ growth rate and growth of *Hericium erinaceus* strains in different proportions of ginger straw medium.

Strains	Supplemented Proportion of Ginger (%)	Growth Rate (mm d^−1^)	Color	Marginal Regularity	Density	Growth
H4	0	5.14 ± 0.03 ^a^	white	neat	dense	+++
	10	4.05 ± 0.23 ^b^	white	relatively neat	dense	++
	15	3.89 ± 0.09 ^b^	white	neat	dense	+++
	20	2.95 ± 0.22 ^c^	white	neat	relatively dense	++
	25	2.72 ± 0.06 ^cd^	white	neat	relatively dense	++
	30	2.68 ± 0.14 ^cd^	white	relatively neat	relatively dense	++
	35	2.51 ± 0.15 ^d^	white	neat	relatively dense	++
	40	2.76 ± 0.1 ^cd^	white	neat	relatively dense	++
H9	0	5.47 ± 0.08 ^a^	white	untidy	dense	++
	10	5.01 ± 0.21 ^b^	white	neat	dense	+++
	15	4.36 ± 0.16 ^c^	white	neat	dense	+++
	20	3.45 ± 0.17 ^d^	white	neat	relatively dense	++
	25	3.04 ± 0.01 ^e^	white	neat	sparse	++
	30	3.37 ± 0.12 ^d^	white	relatively neat	dense	++
	35	2.81 ± 0.11 ^e^	white	neat	sparse	++
	40	2.83 ± 0.09 ^e^	white	relatively neat	sparse	+
H10	0	5.24 ± 0.13 ^a^	snow-white	neat	dense	+++
	10	3.85 ± 0.12 ^b^	snow-white	relatively neat	dense	++
	15	3.97 ± 0.1 ^b^	snow-white	neat	dense	+++
	20	3.05 ± 0.08 ^c^	snow-white	untidy	dense	++
	25	3.05 ± 0.28 ^c^	snow-white	neat	relatively dense	++
	30	2.99 ± 0.16 ^c^	snow-white	relatively neat	dense	++
	35	2.85 ± 0.08 ^cd^	snow-white	neat	relatively dense	++
	40	2.66 ± 0.04 ^d^	ashen	relatively neat	dense	+

Note: “+”—poor growth; “++”—growth is average; “+++”—the growth is better than average. Different lowercase letters indicate significant differences, *p* < 0.05.

**Table 3:** Biological efficiency and commodity traits of the fruiting body of *Pleurotus geesteranus*.

Strain	Proportion of Gradient (%)	Length of Stipe (mm)	Diameter of Cap (mm)	Thickness of Cap (mm)	Water Content (%)
X2	0	52.52 ± 5.11 ^a^	73.02 ± 4.25 ^a^	2.63 ± 0.32 ^bc^	88.16 ± 1.57 ^b^
	25	47.29 ± 2.77 ^bc^	64.37 ± 3.02 ^b^	2.59 ± 0.17 ^bc^	91.59 ± 0.33 ^a^
X3	0	43.07 ± 5.28 ^bc^	58.08 ± 2.71 ^bc^	1.99 ± 0.41 ^d^	90.78 ± 0.57 ^a^
	30	44.25 ± 3.5 ^bc^	59.78 ± 1.37 ^bc^	2.66 ± 0.14 ^bc^	90.5 ± 0.41 ^a^
X4	0	39.79 ± 3.36 ^c^	53.61 ± 0.59 ^cde^	2.78 ± 0.19 ^b^	91.08 ± 0.29 ^a^
	25	39.84 ± 1.32 ^d^	54.52 ± 0.98 ^cd^	2.86 ± 0.11 ^b^	91.13 ± 0.31 ^a^
X5	0	41.88 ± 1.57 ^bc^	74.16 ± 1.87 ^a^	2.27 ± 0.08 ^cd^	91.51 ± 0.16 ^a^
	15	46.54 ± 0.09 ^b^	76.93 ± 1.88 ^a^	2.62 ± 0.14 ^bc^	91.44 ± 0.03 ^a^
X6	0	44.5 ± 3.99 ^bc^	43.46 ± 0.98 ^f^	2.68 ± 0.33 ^bc^	90.73 ± 0.43 ^a^
	20	51.7 ± 0.9 ^b^	51.53 ± 5.46 ^e^	2.99 ± 0.11 ^a^	91.62 ± 0.07 ^a^

Note: Different lowercase letters indicate significant differences, *p* < 0.05.

**Table 4:** Comparison of essential amino acids in *Pleurotus geesteranus* strains (%).

Amino Acid	Strains										Egg [43]	FAO/WHO [41]
	X2(0)	X2(25)	X3(0)	X3(30)	X4(0)	X4(25)	X5(0)	X5(15)	X6(0)	X6(20)		
Thr	4.65 ± 0.18 ^de^	4.94 ± 0.54 ^b^	5.04 ± 0.21 ^ab^	4.69 ± 0.44 ^d^	4.54 ± 0.51 ^e^	4.11 ± 0.56 ^f^	4.61 ± 0.08 ^de^	4.72 ± 0.14 ^c^	5.15 ± 0.02 ^a^	4.76 ± 0.12 ^bc^	5.1	4
Val	5.73 ± 0.51 ^abc^	5.63 ± 0.21 ^abc^	5.97 ± 0.13 ^ab^	6.12 ± 0.47 ^a^	5.41 ± 0.19 ^bc^	5.26 ± 0.11 ^c^	5.91 ± 0.33 ^ab^	5.57 ± 0.22 ^abc^	5.36 ± 0.32 ^bc^	5.58 ± 0.41 ^abc^	7.3	5
Met+Cys	1.61 ± 0.48 ^a^	1.59 ± 0.03 ^a^	1.65 ± 0.21 ^a^	1.76 ± 0.11 ^a^	1.52 ± 0.52 ^a^	1.58 ± 0.13 ^a^	1.59 ± 0.04 ^a^	1.61 ± 0.15 ^a^	1.77 ± 0.04 ^a^	1.91 ± 0.33 ^a^	5.5	3.5
Ile	5.64 ± 0.56 ^ab^	5.73 ± 0.49 ^ab^	5.88 ± 0.18 ^ab^	6.15 ± 0.28 ^a^	4.55 ± 0.78 ^c^	5.27 ± 0.13 ^b^	5.38 ± 0.19 ^ab^	5.58 ± 0.53 ^ab^	5.85 ± 0.33 ^ab^	5.54 ± 0.26 ^ab^	5.5	3.5
Leu	7.61 ± 0.98 ^abc^	8.37 ± 1.81 ^a^	7.61 ± 0.19 ^abc^	8.08 ± 0.85 ^ab^	6.43 ± 0.29 ^c^	6.58 ± 0.52 ^c^	7.04 ± 0.07 ^abc^	6.79 ± 0.17 ^bc^	7.15 ± 0.59 ^abc^	7.01 ± 0.01 ^abc^	8.8	7
Phe+Tyr	8.11 ± 0.88 ^bcd^	8.25 ± 0.17 ^bcd^	8.98 ± 0.34 ^a^	9.11 ± 0.21 ^a^	7.63 ± 0.28 ^d^	8.02 ± 0.06 ^cd^	8.77 ± 0.29 ^ab^	8.23 ± 0.29 ^bcd^	8.96 ± 0.33 ^a^	8.57 ± 0.34 ^abc^	10	6
Lys	5.82 ± 0.75 ^abc^	4.86 ± 0.6 ^d^	5.86 ± 0.21 ^ab^	6.05 ± 0.15 ^a^	5.02 ± 0.01 ^cd^	5.6 ± 0.4 ^abcd^	5.38 ± 0.11 ^abcd^	5.14 ± 0.55 ^bcd^	5.44 ± 0.11 ^abcd^	5.08 ± 0.57 ^bcd^	6.4	5.5
Total	39.44 ± 1.01 ^bc^	39.26 ± 0.82 ^c^	40.85 ± 0.48 ^ab^	41.86 ± 0.27 ^a^	34.98 ± 0.27 ^f^	36.35 ± 0.47 ^ef^	38.54 ± 0.61 ^cd^	37.52 ± 0.33 ^de^	39.52 ± 0.3 ^bc^	38.35 ± 1.53 ^cd^	49.7	35

Note: Different lowercase letters indicate significant differences, *p* < 0.05.

**Table 5:** Amino acid score of the fruiting body.

Amino Acid	Strains									
	X2(0)	X2(25)	X3(0)	X3(30)	X4(0)	X4(25)	X5(0)	X5(15)	X6(0)	X6(20)
Thr	1.16 ± 0.05 ^ab^	1.14 ± 0.13 ^a^	1.26 ± 0.05 ^a^	1.17 ± 0.11 ^ab^	1.14 ± 0.13 ^ab^	1.03 ± 0.14 ^b^	1.15 ± 0.02 ^ab^	1.18 ± 0.04 ^ab^	1.29 ± 0.01 ^a^	1.19 ± 0.03 ^ab^
Val	1.15 ± 0.11 ^abc^	1.13 ± 0.04 ^abc^	1.19 ± 0.03 ^ab^	1.22 ± 0.09 ^a^	1.08 ± 0.0 ^bc^	1.05 ± 0.02 ^c^	1.18 ± 0.07 ^ab^	1.11 ± 0.04 ^abc^	1.07 ± 0.06 ^bc^	1.12 ± 0.08 ^abc^
Met+Cys	0.46 ± 0.14 ^a^	0.46 ± 0.01 ^a^	0.47 ± 0.06 ^a^	0.5 ± 0.03 ^a^	0.43 ± 0.15 ^a^	0.45 ± 0.04 ^a^	0.45 ± 0.01 ^a^	0.46 ± 0.04 ^a^	0.51 ± 0.01 ^a^	0.55 ± 0.09 ^a^
Ile	1.61 ± 0.16 ^ab^	1.64 ± 0.14 ^ab^	1.68 ± 0.05 ^ab^	1.76 ± 0.08 ^a^	1.3 ± 0.22 ^c^	1.51 ± 0.04 ^b^	1.54 ± 0.05 ^ab^	1.59 ± 0.15 ^ab^	1.67 ± 0.09 ^ab^	1.58 ± 0.07 ^ab^
Leu	1.09 ± 0.14 ^abc^	1.19 ± 0.26 ^a^	1.08 ± 0.03 ^abc^	1.15 ± 0.12 ^ab^	0.92 ± 0.04 ^c^	0.94 ± 0.07 ^c^	1.01 ± 0.01 ^abc^	0.97 ± 0.02 ^bc^	1.02 ± 0.09 ^abc^	1.01 ± 0.01 ^abc^
Phe+Tyr	1.35 ± 0.15 ^bcd^	1.38 ± 0.03 ^bcd^	1.5 ± 0.06 ^a^	1.52 ± 0.03 ^a^	1.27 ± 0.05 ^d^	1.34 ± 0.01 ^cd^	1.46 ± 0.05 ^ab^	1.37 ± 0.05 ^bcd^	1.49 ± 0.06 ^a^	1.43 ± 0.06 ^abc^
Lys	1.06 ± 0.14 ^abc^	0.88 ± 0.11 ^d^	1.06 ± 0.04 ^ab^	1.11 ± 0.02 ^a^	0.91 ± 0.02 ^cd^	1.02 ± 0.07 ^abcd^	0.98 ± 0.02 ^abcd^	0.94 ± 0.01 ^bcd^	0.99 ± 0.02 ^abcd^	0.92 ± 0.11 ^bcd^
Total	1.13 ± 0.03 ^bc^	1.12 ± 0.02 ^c^	1.17 ± 0.01 ^ab^	1.19 ± 0.01 ^a^	1 ± 0.03 ^f^	1.04 ± 0.01 ^ef^	1.1 ± 0.02 ^cd^	1.07 ± 0.01 ^de^	1.13 ± 0.01 ^bc^	1.1 ± 0.04 ^cd^

Note: Different lowercase letters indicate significant differences, *p* < 0.05.

**Table 6:** Chemical score (cs) and essential amino acid index of the fruiting body.

Amino Acid	Strains									
	X2(0)	X2(25)	X3(0)	X3(30)	X4(0)	X4(25)	X5(0)	X5(15)	X6(0)	X6(20)
Thr	0.91 ± 0.04 ^ab^	0.97 ± 0.11 ^a^	0.99 ± 0.04 ^a^	0.92 ± 0.09 ^ab^	0.89 ± 0.09 ^ab^	0.81 ± 0.11 ^b^	0.9 ± 0.02 ^ab^	0.93 ± 0.03 ^ab^	1.01 ± 0.01 ^a^	0.93 ± 0.02 ^ab^
Val	0.79 ± 0.07 ^abc^	0.77 ± 0.03 ^abc^	0.82 ± 0.02 ^ab^	0.84 ± 0.06 ^a^	0.74 ± 0.03 ^bc^	0.72 ± 0.01 ^c^	0.81 ± 0.05 ^ab^	0.76 ± 0.03 ^abc^	0.73 ± 0.04 ^bc^	0.76 ± 0.06 ^abc^
Met+Cys	0.29 ± 0.09 ^a^	0.29 ± 0.01 ^a^	0.3 ± 0.04 ^a^	0.32 ± 0.02 ^a^	0.28 ± 0.09 ^a^	0.29 ± 0.03 ^a^	0.29 ± 0.01 ^a^	0.29 ± 0.03 ^a^	0.32 ± 0.01 ^a^	0.35 ± 0.06 ^a^
Ile	1.03 ± 0.11 ^ab^	1.04 ± 0.09 ^ab^	1.07 ± 0.03 ^ab^	1.12 ± 0.05 ^a^	0.83 ± 0.14 ^c^	0.96 ± 0.02 ^b^	0.98 ± 0.03 ^ab^	1.02 ± 0.09 ^ab^	1.06 ± 0.06 ^ab^	1.01 ± 0.05 ^ab^
Leu	0.86 ± 0.11 ^abc^	0.95 ± 0.21 ^a^	0.86 ± 0.02 ^abc^	0.92 ± 0.1 ^ab^	0.73 ± 0.03 ^c^	0.75 ± 0.06 ^c^	0.8 ± 0.01 ^abc^	0.77 ± 0.02 ^bc^	0.81 ± 0.07 ^abc^	0.8 ± 0.01 ^abc^
Phe+Tyr	0.81 ± 0.09 ^bcd^	0.83 ± 0.02 ^bcd^	0.89 ± 0.03 ^a^	0.91 ± 0.02 ^a^	0.76 ± 0.03 ^d^	0.8 ± 0.1 ^cd^	0.88 ± 0.03 ^ab^	0.82 ± 0.03 ^bcd^	0.89 ± 0.03 ^a^	0.86 ± 0.03 ^abc^
Lys	0.91 ± 0.12 ^abc^	0.76 ± 0.1 ^d^	0.92 ± 0.03 ^ab^	0.95 ± 0.02 ^a^	0.78 ± 0.01 ^cd^	0.88 ± 0.06 ^abcd^	0.84 ± 0.02 ^abcd^	0.8 ± 0.09 ^bcd^	0.85 ± 0.02 ^abcd^	0.79 ± 0.09 ^bcd^
Total	0.79 ± 0.02 ^bc^	0.79 ± 0.02 ^c^	0.82 ± 0.01 ^ab^	0.84 ± 0.1 ^a^	0.7 ± 0.02 ^f^	0.73 ± 0.1 ^ef^	0.76 ± 0.01 ^cd^	0.75 ± 0.01 ^de^	0.76 ± 0.01 ^bc^	0.77 ± 0.03 ^cd^
EAAI	1.3 ± 0.01 ^bcd^	1.3 ± 0.01 ^bcd^	1.31 ± 0.01 ^ab^	1.32 ± 0.01 ^a^	1.28 ± 0.01 ^f^	1.29 ± 0.01 ^ef^	1.3 ± 0.01 ^cd^	1.3 ± 0.01 ^de^	1.31 ± 0.01 ^bc^	1.29 ± 0.01 ^cd^

Note: different lowercase letters indicate significant differences, *p* < 0.05.

**Table 7:** Biological efficiency and commodity traits of the fruiting body of *Hericium erinaceus*.

Strains	Proportion of Gradient (%)	Diameter of Cap (mm)	Water Content (%)
H4	0	67.27 ± 1.62 ^a^	90.5 ± 0.54 ^ab^
	15	72.76 ± 4.58 ^a^	90.52 ± 0.29 ^ab^
H9	0	68.97 ± 3.89 ^a^	89.91 ± 0.54 ^bc^
	15	72.58 ± 1.28 ^b^	89.4 ± 0.11 ^c^
H10	0	71.95 ± 0.69 ^a^	90.52 ± 0.11 ^ab^
	15	56.66 ± 3.78 ^b^	90.68 ± 0.29 ^a^

Note: different lowercase letters indicate significant differences, *p* < 0.05.

**Table 8:** Comparison of essential amino acids in *Hericium erinaceus* strains (%).

Amino Acid	Strains						Egg [43]	FAO/WHO [41]
	H4(0)	H4(15)	H9(0)	H9(15)	H10(0)	H10(15)		
Thr	5.6 ± 0.58 ^a^	5.27 ± 0.16 ^a^	5.54 ± 0.76 ^a^	5.65 ± 0.58 ^a^	5.29 ± 0.37 ^a^	5.28 ± 0.17 ^a^	5.1	4
Val	10.19 ± 0.83 ^a^	9.57 ± 0.19 ^ab^	9.71 ± 0.69 ^a^	9.26 ± 0.79 ^ab^	8.41 ± 0.24 ^b^	9.36 ± 0.77 ^ab^	7.3	5
Met+Cys	1.47 ± 0.28 ^a^	2.73 ± 0.98 ^a^	1.59 ± 0.26 ^a^	1.49 ± 0.07 ^a^	1.73 ± 0.35 ^a^	2.06 ± 1.4 ^a^	5.5	3.5
Ile	8.28 ± 1.04 ^ab^	8.98 ± 1.01 ^a^	8.26 ± 0.27 ^ab^	7.19 ± 0.32 ^bc^	5.97 ± 0.69 ^c^	7.06 ± 0.54 ^bc^	5.5	3.5
Leu	9.98 ± 0.61 ^a^	9.49 ± 0.71 ^a^	9.87 ± 0.32 ^a^	9.5 ± 0.23 ^a^	9.3 ± 0.02 ^a^	9.25 ± 0.54 ^a^	8.8	7
Phe+Tyr	12.45 ± 0.18 ^a^	10.57 ± 0.68 ^bc^	11.44 ± 0.66 ^b^	10.89 ± 0.72 ^b^	9.62 ± 0.47 ^c^	10.94 ± 0.41 ^b^	10	6
Lys	4.64 ± 0.94 ^a^	5.38 ± 0.33 ^a^	5.72 ± 1.39 ^a^	5.86 ± 0.64 ^a^	5.25 ± 0.29 ^a^	5.64 ± 0.27 ^a^	6.4	5.5
Total	52.56 ± 0.87 ^a^	51.81 ± 2.5 ^ab^	51.99 ± 2.07 ^ab^	49.69 ± 1.25 ^ab^	45.42 ± 0.64 ^b^	49.45 ± 0.74 ^c^	49.7	35

Note: Different lowercase letters indicate significant differences, *p* < 0.05.

**Table 9:** Amino acid score of the fruiting body of *Hericum erinaceus*.

Amino Acid	Strains					
	H4(0)	H4(15)	H9(0)	H9(15)	H10(0)	H10(15)
Thr	1.41 ± 0.15 ^a^	1.32 ± 0.04 ^a^	1.39 ± 0.19 ^a^	1.41 ± 0.14 ^a^	1.32 ± 0.09 ^a^	1.32 ± 0.04 ^a^
Val	2.04 ± 0.17 ^a^	1.91 ± 0.04 ^ab^	1.94 ± 0.14 ^a^	1.85 ± 0.16 ^ab^	1.68 ± 0.05 ^b^	1.87 ± 0.15 ^ab^
Met+Cys	0.42 ± 0.08 ^a^	0.78 ± 0.28 ^a^	0.45 ± 0.07 ^a^	0.43 ± 0.02 ^a^	0.49 ± 0.1 ^a^	0.59 ± 0.04 ^a^
Ile	2.36 ± 0.3 ^ab^	2.57 ± 0.29 ^a^	2.36 ± 0.07 ^ab^	2.06 ± 0.09 ^bc^	1.71 ± 0.19 ^c^	2.02 ± 0.15 ^bc^
Leu	1.43 ± 0.09 ^a^	1.36 ± 0.11 ^a^	1.41 ± 0.05 ^a^	1.36 ± 0.03 ^a^	1.33 ± 0.01 ^a^	1.32 ± 0.08 ^a^
Phe+Tyr	2.08 ± 0.03 ^a^	1.7+ ± 0.11 ^bc^	1.91 ± 0.11 ^b^	1.82 ± 0.12 ^b^	1.61 ± 0.07 ^c^	1.83 ± 0.07 ^b^
Lys	0.84 ± 0.17 ^a^	0.98 ± 0.06 ^a^	1.04 ± 0.25 ^a^	1.07 ± 0.12 ^a^	0.95 ± 0.05 ^a^	1.06 ± 0.05 ^a^
Total	1.51 ± 0.03 ^a^	1.48 ± 0.07 ^ab^	1.49 ± 0.06 ^ab^	1.42 ± 0.04 ^ab^	1.29 ± 0.02 ^c^	1.41 ± 0.02 ^b^

Note: Different lowercase letters indicate significant differences, *p* < 0.05.

**Table 10:** Chemical score (cs) and essential amino acid index of the fruiting body of *Hericum erinaceus*.

Amino Acid	Strains					
	H4(0)	H4(15)	H9(0)	H9(15)	H10(0)	H10(15)
Thr	1.09 ± 0.11 ^a^	1.03 ± 0.03 ^a^	1.09 ± 0.15 ^a^	1.11 ± 0.11 ^a^	1.04 ± 0.07 ^a^	1.04 ± 0.04 ^a^
Val	1.39 ± 0.11 ^a^	1.31 ± 0.03 ^ab^	1.33 ± 0.09 ^a^	1.27 ± 0.11 ^ab^	1.15 ± 0.03 ^b^	1.29 ± 0.11 ^ab^
Met+Cys	0.27 ± 0.05 ^a^	0.5 ± 0.18 ^a^	0.29 ± 0.05 ^a^	0.27 ± 0.02 ^a^	0.31 ± 0.06 ^a^	0.37 ± 0.05 ^a^
Ile	1.51 ± 0.19 ^ab^	1.63 ± 0.18 ^a^	1.51 ± 0.05 ^ab^	1.31 ± 0.06 ^bc^	1.08 ± 0.13 ^c^	1.28 ± 0.09 ^bc^
Leu	1.13 ± 0.07 ^a^	1.08 ± 0.08 ^a^	1.12 ± 0.04 ^a^	1.08 ± 0.03 ^a^	1.06 ± 0.01 ^a^	1.05 ± 0.06 ^a^
Phe+Tyr	1.24 ± 0.02 ^a^	1.06 ± 0.07 ^bc^	1.14 ± 0.07 ^b^	1.09 ± 0.07 ^b^	0.96 ± 0.05 ^c^	1.09 ± 0.04 ^b^
Lys	0.93 ± 0.45 ^a^	0.84 ± 0.05 ^a^	0.89 ± 0.22 ^a^	0.92 ± 0.1 ^a^	0.82 ± 0.05 ^a^	0.88 ± 0.04 ^a^
Total	1.06 ± 0.02 ^a^	1.04 ± 0.05 ^ab^	1.05 ± 0.04 ^ab^	1 ± 0.03 ^ab^	0.91 ± 0.02 ^c^	0.99 ± 0.01 **^b^**
EAAI	1.36 ± 0.01 ^ab^	1.36 ± 0.01 ^a^	1.36 ± 0.01 ^ab^	1.35 ± 0.01 ^ab^	1.33 ± 0.01 **^c^**	1.35 ± 0.01 **^b^**

Note: Different lowercase letters indicate significant differences, *p* < 0.05.

**Table 11:** The average membership function value of the fruiting body of *Pleurotus geesteranus*.

Item	Fruiting Body									
	X2(0)	X2(25)	X3(0)	X3(30)	X4(0)	X4(25)	X5(0)	X5(15)	X6(0)	X6(20)
Total sugar	0.5	0.52	0.44	0.59	0.6	0.6	0.42	0.41	0.6	0.49
Crude fat	0.4	0.54	0.41	0.61	0.39	0.45	0.39	0.63	0.39	0.34
Crude protein	0.42	0.48	0.61	0.66	0.41	0.37	0.39	0.52	0.58	0.49
Reducing sugar	0.38	0.62	0.48	0.49	0.42	0.45	0.55	0.48	0.56	0.67
Total antioxidant capacity	0.52	0.67	0.46	0.56	0.56	0.67	0.55	0.59	0.4	0.42
Biological efficiency	0.61	0.57	0.6	0.39	0.44	0.43	0.58	0.38	0.64	0.34
Stipe lengths	0.47	0.36	0.5	0.42	0.5	0.49	0.61	0.42	0.44	0.48
Cap diameters	0.47	0.48	0.34	0.61	0.51	0.36	0.59	0.54	0.42	0.66
Thickness of the Cap	0.46	0.43	0.42	0.35	0.51	0.6	0.38	0.35	0.34	0.62
Water content	0.37	0.59	0.57	0.35	0.54	0.55	0.42	0.41	0.62	0.61
Ash content	0.57	0.48	0.5	0.53	0.57	0.47	0.5	0.55	0.4	0.53
crude fiber	0.49	0.5	0.44	0.45	0.55	0.51	0.48	0.33	0.46	0.51
Total amino acids	0.53	0.55	0.46	0.49	0.41	0.51	0.45	0.45	0.52	0.4
Average membership function value	0.48	0.52	0.48	0.5	0.49	0.5	0.48	0.47	0.5	0.5
The average membership function value of fruiting bodies cultivated with ginger straw: 0.497					The average membership function value of fruiting bodies cultivated in cottonseed hulls: 0.484					

**Table 12:** The average membership function value of the fruiting body of *Hericium erinaceus*.

Item	Fruiting Body					
	H4(0)	H4(15)	H9(0)	H9(15)	H10(0)	H10(15)
Total sugar	0.5	0.6	0.48	0.44	0.53	0.470.52
Crude fat	0.52	0.53	0.47	0.56	0.4	0.42
Crude protein	0.51	0.58	0.44	0.33	0.5	0.39
Reducing sugar	0.44	0.57	0.44	0.55	0.4	0.52
Total antioxidant capacity	0.56	0.47	0.33	0.33	0.42	0.45
Biological efficiency	0.48	0.55	0.37	0.5	0.43	0.4
Diameter of Fruiting Body	0.44	0.43	0.66	0.34	0.66	0.39
Water content	0.39	0.55	0.66	0.4	0.41	0.48
Ash content	0.58	0.48	0.49	0.52	0.5	0.56
crude fiber	0.45	0.55	0.4	0.5	0.63	0.54
Total amino acids	0.58	0.46	0.52	0.5	0.42	0.54
Average membership function value	0.49	0.52	0.48	0.45	0.48	0.47
The average membership function value of fruiting bodies cultivated with ginger straw: 0.481			The average membership function value of fruiting bodies cultivated in cottonseed hulls: 0.484			

## Data Availability

The original contributions presented in this study are included in the article/Supplementary Materials. Further inquiries can be directed to the corresponding authors.

## Supplementary Materials

The following supporting information can be downloaded at https://www.mdpi.com/article/10.3390/foods15050898/s1. Table S1: The mycelial growth rate and growth vigor of *Pleurotus geesteranus* strains on PDA medium. Table S2: The mycelial growth rate and growth vigor of *Hericium erinaceus* strains on PDA medium. Table S3: Mycelial growth rate and growth vigor of *Pleurotus geesteranus* in conventional cottonseed hull medium. Table S4: Mycelial growth rate and growth vigor of *Hericium erinaceus* in conventional cottonseed hull medium.

## Appendix A

**Table A1:** The strains used in this study.

Edible Fungi	Strain Name in This Study	The Original Name of Strains	Sources	Artificial Cultivation Substrate	Growth Environment
*Pleurotus geesteranus*	X1	g27	Department of Mycology, College of Plant Protection, Shandong Agricultural University	Stock cultures were maintained on PDA medium, while cultivation was performed using cottonseed hull-based substrate.	Stock cultures were stored at 4 °C, and mycelial cultivation was performed at 25 °C in the dark.
	X2	P4+1	Department of Mycology, College of Plant Protection, Shandong Agricultural University		
	X3	g35	Department of Mycology, College of Plant Protection, Shandong Agricultural University	Stock cultures were maintained on PDA medium, while cultivation was performed using cottonseed hull-based substrate.	Stock cultures were stored at 4 °C, and mycelial cultivation was performed at 25 °C in the dark.
	X4	P3	Department of Mycology, College of Plant Protection, Shandong Agricultural University		
	X5	Plg2	Department of Mycology, College of Plant Protection, Shandong Agricultural University		
	X6	X155	Department of Mycology, College of Plant Protection, Shandong Agricultural University		
	X7	g23	Department of Mycology, College of Plant Protection, Shandong Agricultural University		
*Hericium erinaceus*	H1	*Hericium erinaceus*—Jixi	Department of Mycology, College of Plant Protection, Shandong Agricultural University	Stock cultures were maintained on PDA medium, while cultivation was performed using cottonseed hull-based substrate.	Stock cultures were stored at 4 °C, and mycelial cultivation was performed at 25 °C in the dark.
	H2	*Hericium erinaceus*—Mudanjiang	Department of Mycology, College of Plant Protection, Shandong Agricultural University		
	H3	CCMSSC 02820	The Institute of Agricultural Resources and Regional Planning, Chinese Academy of Agricultural Sciences		
	H4	CCMSSC 02821	The Institute of Agricultural Resources and Regional Planning, Chinese Academy of Agricultural Sciences		
	H5	CCMSSC 02822	The Institute of Agricultural Resources and Regional Planning, Chinese Academy of Agricultural Sciences		
	H6	*Hericium erinaceus*-1	Jilin Agricultural University		
	H7	*Hericium erinaceus*-2	Jilin Agricultural University		
	H8	*Hericium erinaceus*-5	Jilin Agricultural University		
	H9	2607	Jilin Agricultural University		
	H10	2608	Jilin Agricultural University		
	H11	2614	Jilin Agricultural University		
	H12	2615	Jilin Agricultural University		

**Table A2:** Evaluation criterion for mycelial growth vigor.

Character	Criterion	Character	Criterion
Mycelial Color	snow-white	Mycelial Growth Vigor	excellent
	white		good
	ashen		ordinary
Mycelial Edge Regularity	neat		weak
	relatively neat	Mycelial Density	dense
	untidy		relatively dense
			sparse

**Table A3:** Physicochemical properties of ginger straw.

Index	Content	Unit	Test Method
Lignin	209.14 ± 1.35	mg g^−1^	Micro-method
Cellulose	338.9 ± 5.42	mg g^−1^	Micro-method
Hemicellulose	111.52 ± 3.68	mg g^−1^	Micro-method
Crude fat	1.3 ± 0.14	%	GB/T 6433-2006 [74]
crude protein	13.98 ± 0.43	%	GB/T 6432-2016 [75]
Volatile oil	0	mL/100 g	GB/T 30385-2013 [76]
C element	36.54 ± 2.77	%	Elemental analyzer method
N element	1.74 ± 0.16	%	Elemental analyzer method
K^+^	33,943.99 ± 35.62	mg kg^−1^	Ion chromatography
Ash	14.2 ± 0.23	%	GB/5009.4-2016 [77]
pH	7.65 ± 0.14	/	pH meter method
Arsenic (As)	0.219 ± 0.06	mg kg^−1^	GB/5009.268-2016 [78]
Cadmium (Cd)	0.046 ± 0.003	mg kg^−1^	GB/5009.268-2016
Mercury (Hg)	0.069 ± 0.001	mg kg^−1^	GB/5009.268-2016
Lead (Pb)	0.515 ± 0.01	mg kg^−1^	GB/5009.268-2016
Chromium (Cr)	0.46 ± 0.005	mg kg^−1^	GB/5009.268-2016
DDT	Not detected (<0.01)	mg kg^−1^	GB 23200.113-2018 [79]
Dieldrin	Not detected (<0.01)	mg kg^−1^	GB 23200.113-2018
Difenoconazole	Not detected (<0.01)	mg kg^−1^	GB 23200.113-2018
Chlorpyrifos	Not detected (<0.01)	mg kg^−1^	GB 23200.113-2018
Diazinon	Not detected (<0.01)	mg kg^−1^	GB 23200.113-2018
Cyfluthrin and beta-cyfluthrin	Not detected (<0.01)	mg kg^−1^	GB 23200.113-2018
Hexaconazole	Not detected (<0.01)	mg kg^−1^	GB 23200.113-2018
Fenpropathrin	Not detected (<0.01)	mg kg^−1^	GB 23200.113-2018
Bifenthrin	Not detected (<0.01)	mg kg^−1^	GB 23200.113-2018
Azoxystrobin	Not detected (<0.01)	mg kg^−1^	GB 23200.113-2018
Pyrimethanil	Not detected (<0.01)	mg kg^−1^	GB 23200.113-2018
Deltamethrin	Not detected (<0.01)	mg kg^−1^	GB 23200.113-2018
Fenthion	Not detected (<0.01)	mg kg^−1^	GB 23200.113-2018
Dichlorvos	Not detected (<0.01)	mg kg^−1^	GB 23200.113-2018
Fonofos	Not detected (<0.01)	mg kg^−1^	GB 23200.113-2018
Parathion	Not detected (<0.01)	mg kg^−1^	GB 23200.113-2018
Methamidophos	Not detected (<0.01)	mg kg^−1^	GB 23200.113-2018
Phorate	Not detected (<0.01)	mg kg^−1^	GB 23200.113-2018
Methyl parathion	Not detected (<0.01)	mg kg^−1^	GB 23200.113-2018
Methyl isothiophos	Not detected (<0.01)	mg kg^−1^	GB 23200.113-2018
Monocrotophos	Not detected (<0.01)	mg kg^−1^	GB 23200.113-2018
Pirimicarb	Not detected (<0.01)	mg kg^−1^	GB 23200.113-2018
Dimethoate	Not detected (<0.01)	mg kg^−1^	GB 23200.113-2018
Phosphamidon	Not detected (<0.01)	mg kg^−1^	GB 23200.113-2018
Phosfolan	Not detected (<0.01)	mg kg^−1^	GB 23200.113-2018
Chloroneb	Not detected (<0.01)	mg kg^−1^	GB 23200.113-2018
Isazofos	Not detected (<0.01)	mg kg^−1^	GB 23200.113-2018
Cyprodinil	Not detected (<0.01)	mg kg^−1^	GB 23200.113-2018
Ethoprophos	Not detected (<0.01)	mg kg^−1^	GB 23200.113-2018
Oxyfluorfen	Not detected (<0.01)	mg kg^−1^	GB 23200.113-2018
Dicofol	Not detected (<0.01)	mg kg^−1^	GB 23200.113-2018
Tetrachlorvinphos	Not detected (<0.01)	mg kg^−1^	GB 23200.113-2018
Fenitrothion	Not detected (<0.01)	mg kg^−1^	GB 23200.113-2018
Methidathion	Not detected (<0.01)	mg kg^−1^	GB 23200.113-2018
Isocarbophos	Not detected (<0.01)	mg kg^−1^	GB 23200.113-2018
Mevinphos	Not detected (<0.01)	mg kg^−1^	GB 23200.113-2018
Omethoate	Not detected (<0.01)	mg kg^−1^	GB 23200.113-2018
Acephate	Not detected (<0.01)	mg kg^−1^	GB 23200.113-2018
Coumaphos	Not detected (<0.01)	mg kg^−1^	GB 23200.113-2018
Sulfotep	Not detected (<0.01)	mg kg^−1^	GB 23200.113-2018
Aldrin	Not detected (<0.01)	mg kg^−1^	GB 23200.113-2018
Pyraclostrobin	Not detected (<0.01)	mg kg^−1^	GB 23200.113-2018
Tebufenozide	Not detected (<0.01)	mg kg^−1^	GB 23200.113-2018
Boscalid	Not detected (<0.01)	mg kg^−1^	GB 23200.113-2018
Carbendazim	Not detected (<0.01)	mg kg^−1^	GB 23200.113-2018
Thiacloprid	Not detected (<0.01)	mg kg^−1^	GB 23200.113-2018
Buprofezin	Not detected (<0.01)	mg kg^−1^	GB 23200.113-2018
Tebuconazole	Not detected (<0.01)	mg kg^−1^	GB 23200.113-2018
Indoxacarb	Not detected (<0.01)	mg kg^−1^	GB 23200.113-2018
Phoxim	Not detected (<0.01)	mg kg^−1^	GB 23200.113-2018
Clothianidin	Not detected (<0.01)	mg kg^−1^	GB 23200.113-2018
Demeton	Not detected (<0.01)	mg kg^−1^	GB 23200.113-2018
Chlorsulfuron	Not detected (<0.01)	mg kg^−1^	GB 23200.113-2018
Cadusafos	Not detected (<0.01)	mg kg^−1^	GB 23200.113-2018
Imidacloprid	Not detected (<0.01)	mg kg^−1^	GB 23200.113-2018
Trichlorfon	Not detected (<0.01)	mg kg^−1^	GB 23200.113-2018
Acetamiprid	Not detected (<0.01)	mg kg^−1^	GB 23200.113-2018
Emamectin benzoate	Not detected (<0.01)	mg kg^−1^	GB 23200.113-2018
Forchlorfenuron	Not detected (<0.01)	mg kg^−1^	GB 23200.113-2018
Fenamiphos	Not detected (<0.01)	mg kg^−1^	GB 23200.113-2018
Erbon	Not detected (<0.01)	mg kg^−1^	GB 23200.113-2018
Chlorfenapyr	Not detected (<0.01)	mg kg^−1^	GB 23200.113-2018
Glyphosate	Not detected (<0.01)	mg kg^−1^	GB 23200.113-2018
Carbofuran	Not detected (<0.01)	mg kg^−1^	GB 23200.113-2018
Methyl phosfolan	Not detected (<0.01)	mg kg^−1^	GB 23200.113-2018
Chlordane (sum of cis-chlordane and trans-chlordane)	Not detected (<0.01)	mg kg^−1^	GB 23200.113-2018
Mirex	Not detected (<0.01)	mg kg^−1^	GB 23200.113-2018
Heptachlor	Not detected (<0.01)	mg kg^−1^	GB 23200.113-2018
Endrin	Not detected (<0.01)	mg kg^−1^	GB 23200.113-2018
o,p′-DDD	Not detected (<0.01)	mg kg^−1^	GB 23200.113-2018
o,p′-DDE	Not detected (<0.01)	mg kg^−1^	GB 23200.113-2018
o,p′-DDT	Not detected (<0.01)	mg kg^−1^	GB 23200.113-2018
p,p′-DDD	Not detected (<0.01)	mg kg^−1^	GB 23200.113-2018
p,p′-DDD	Not detected (<0.01)	mg kg^−1^	GB 23200.113-2018
p,p′-DDD	Not detected (<0.01)	mg kg^−1^	GB 23200.113-2018
β-HCH	Not detected (<0.01)	mg kg^−1^	GB 23200.113-2018
δ-HCH	Not detected (<0.01)	mg kg^−1^	GB 23200.113-2018
Lindane (γ-HCH)	Not detected (<0.01)	mg kg^−1^	GB 23200.113-2018
Fipronil	Not detected (<0.01)	mg kg^−1^	GB 23200.113-2018
Flusilazole	Not detected (<0.01)	mg kg^−1^	GB 23200.113-2018
Epoxiconazole	Not detected (<0.01)	mg kg^−1^	GB 23200.113-2018
Trifluralin	Not detected (<0.01)	mg kg^−1^	GB 23200.113-2018
Oxadiazon	Not detected (<0.01)	mg kg^−1^	GB 23200.113-2018
Oxadixyl	Not detected (<0.01)	mg kg^−1^	GB 23200.113-2018
Azinphos-methyl	Not detected (<0.01)	mg kg^−1^	GB 23200.113-2018
Fenthion sulfoxide	Not detected (<0.01)	mg kg^−1^	GB 23200.113-2018
Fenthion sulfone	Not detected (<0.01)	mg kg^−1^	GB 23200.113-2018
Propham	Not detected (<0.01)	mg kg^−1^	GB 23200.113-2018
Mefenacet	Not detected (<0.01)	mg kg^−1^	GB 23200.113-2018
Benalaxyl	Not detected (<0.01)	mg kg^−1^	GB 23200.113-2018
Flufenacet	Not detected (<0.01)	mg kg^−1^	GB 23200.113-2018
Pretilachlor	Not detected (<0.01)	mg kg^−1^	GB 23200.113-2018
Propaphos	Not detected (<0.01)	mg kg^−1^	GB 23200.113-2018
Propiconazole	Not detected (<0.01)	mg kg^−1^	GB 23200.113-2018
Fenbuconazole	Not detected (<0.01)	mg kg^−1^	GB 23200.113-2018
Fenothiocarb	Not detected (<0.01)	mg kg^−1^	GB 23200.113-2018
Chlorobenzilate	Not detected (<0.01)	mg kg^−1^	GB 23200.113-2018
Pyridaben	Not detected (<0.01)	mg kg^−1^	GB 23200.113-2018
fipronil	Not detected (<0.01)	mg kg^−1^	GB 23200.113-2018
