# Supplementary material for: Impact of Ginger Straw on Cultivation and Quality of Pleurotus geesteranus and Hericium erinaceus
Source: Foods. 2026 Mar 5;15(5):898. doi: 10.3390/foods15050898 (PMC12985026; doi:10.3390/foods15050898)
Supplement: Supplementary file 1 [file foods-15-00898-s001.zip › foods-4153089-supplementary.pdf]

Screening of Elite Strains of *Pleurotus geesteranus* and *Hericium erinaceus*

1 Screening of superior strains

1.1 Determination of Mycelial Growth Rate and Growth Vigor of Strains on potato-dextrose-agar (PDA) Medium

1.1.1 Determination of Mycelial Growth Rate and Growth Vigor of *Pleurotus geesteranus* Strains on PDA Medium

There were significant differences in the growth rate of different *P. geesteranus* strains on PDA medium. As shown in Table 1, the growth rate of *P. geesteranus* strains ranged from 2.15 to 3.98 mm/d, among which strain X2 had the fastest growth rate, reaching 3.98 mm/d, while strain X7 had the slowest growth rate, only 2.15 mm/d. Differences were also observed in the growth vigor of different strains: strains X2, X3, X4, X5, and X6 were rated as excellent in growth vigor, with white, dense hyphae and neat edges; strains X1 and X7 were rated as good in hyphal growth vigor, in which strain X1 had grayish-white hyphae and strain X7 had irregular hyphal edges.

Table S1 The mycelial growth rate and growth vigor of *Pleurotus geesteranus* strains on PDA medium

Strains	Growth rate（mm/d）	Color	Marginal regularity	Density	Growth
X1	2.54 ± 0.07^{c}	ashen	untidy	dense	++
X2	3.98 ± 0.1^{a}	white	neat	dense	+++
X3	3.75 ± 0.43^{a}	white	neat	dense	+++
X4	3.33 ± 0.07^{b}	white	neat	dense	+++
X5	3.03 ± 0.15^{b}	white	neat	dense	+++
X6	3.25 ± 0.01^{b}	white	neat	dense	+++
X7	2.15 ± 0.09^{c}	white	untidy	relatively dense	++

Note: “+” poor growth; “++” growth is average; “+++” the growth is better than average；Different lowercase letters indicate significant differences, p<0.05

1.1.2 Determination of Mycelial Growth Rate and Growth Vigor of *Hericium erinaceus* Strains on PDA Medium

The mycelial growth rate and growth vigor of different *H. erinaceus* strains on PDA medium were determined. The results showed that there were differences in the growth rate among different strains. As shown in Table 2, the growth rate of *H. erinaceus* strains ranged from 0.83 to 4.71 mm/d, among which strain H4 had the fastest growth rate, reaching 4.71 mm/d, while strain H3 had the slowest growth rate, only 0.83 mm/d. Differences were also observed in the hyphal growth vigor among different strains: except for strains H6 and H11, the hyphal color of the other strains was mostly snow-white or pure white. Notably, the edge regularity of most *H. erinaceus* strains was low. Only strains H4, H9, and H10 exhibited neat mycelial edges, whereas those of the remaining strains were either relatively neat or irregular. The hyphal growth vigor of *H. erinaceus* varied in quality: strains H4, H9, and H10 were rated as excellent in growth vigor; strains H1, H3, H5, H6, H7, H8, and H12 were rated as good; and strains H2 and H11 were rated as average.

Table S2 The mycelial growth rate and growth vigor of *Hericium erinaceus* strains on PDA medium

Strains	Growth rate（mm/d）	Color	Marginal regularity	Density	Growth
H1	3.74 ± 0.09^{c}	snow-white	untidy	dense	++
H2	3.38 ± 0.1^{d}	snow-white	untidy	dense	+
H3	0.83 ± 0.01^{g}	snow-white	untidy	dense	++
H4	4.71 ± 0.31^{a}	white	neat	dense	+++
H5	1.63 ± 0.23^{f}	white	relatively neat	relatively dense	++
H6	3.64 ± 0.16^{cd}	ashen	untidy	dense	++
H7	3.68 ± 0.03^{cd}	white	relatively neat	dense	++
H8	3.67 ± 0.03^{cd}	white	untidy	relatively dense	++
H9	4.11 ± 0.15^{b}	white	neat	dense	+++
H10	4.65 ± 0.09^{a}	white	neat	dense	+++
H11	2.81 ± 0.13^{e}	ashen	relatively neat	relatively dense	+

H12	3.73±0.04 c	white	relatively neat	relatively dense	++

Note: "+" poor growth; "++" growth is average; "+++" the growth is better than average; Different lowercase letters indicate significant differences, $p<0.05$

1.2 Determination of Mycelial Growth Rate and Growth Vigor of Strains on Cultivation Substrate Medium

To further explore the decomposition capacity of different strains on cultivation substrates, the growth characteristics of different strains in cultivation substrates were determined using the conventional cotton seed hull medium formula as the material.

1.2.1 Determination of Mycelial Growth Rate and Growth Vigor of *Pleurotus geesteranus* Strains on Conventional Cotton Seed Hull Medium

There were significant differences in the mycelial growth rate of *P. geesteranus* strains on the conventional cotton seed hull medium, with the growth rate ranging from 3.70 to 4.64 mm/d (Table 3). Among them, strain X2 had the fastest growth rate (4.64 mm/d), while strain X1 had the slowest (3.70 mm/d). No significant difference in growth rate was observed among strains X2, X3, X4, X5, and X6, which had a significantly faster growth rate than strains X1 and X7. In terms of hyphal growth vigor, except for the low edge regularity of strain X1 and the low hyphal density of strain X7, the hyphal color of the other strains was snow-white, with neat edges and high density. Strains X2, X3, X4, X5, and X6 were rated as excellent in hyphal growth vigor, while strains X1 and X7 were rated as good.

Table S3 Mycelial growth rate and growth vigor of *Pleurotus geesteranus* in conventional cottonseed hull medium

Strains	Growth rate（mm/d）	Color	Marginal regularity	Density	Growth
X1	3.7±0.3 b	snow-white	relatively neat	dense	++
X2	4.64±0.2 a	snow-white	neat	dense	+++
X3	4.5±0.1 a	snow-white	neat	dense	+++
X4	4.33±0.05 a	snow-white	neat	dense	+++
X5	4.23±0.21 a	snow-white	neat	dense	+++
X6	4.2±0.05 a	snow-white	neat	dense	+++

X7	3.79±0.18 b	snow-white	neat	relatively dense	++

Note: "+" poor growth; "++" growth is average; "+++" the growth is better than average；Different lowercase letters indicate significant differences, $p<0.05$

1.2.2 Determination of Mycelial Growth Rate and Growth Vigor of *Hericium erinaceus* Strains on Conventional Cotton Seed Hull Medium

There were significant differences in the mycelial growth rate among different *H. erinaceus* strains (Table 4). Strain H4 had the fastest growth rate, reaching 2.60 mm/d, while strain H3 had the slowest, only 1.22 mm/d. Significant differences were also observed in the hyphal color of different strains: strains H1, H2, H3, H4, H6, H8, H9, and H10 had pure white or snow-white hyphae, while strains H5, H7, and H11 had grayish-white hyphae. In terms of hyphal edge regularity, two-thirds of the strains had high edge regularity, and only strains H1, H6, H7, and H8 had relatively neat or irregular edges. There were differences in the hyphal density among different tested *H. erinaceus* strains: strains H1, H2, H3, and H11 had sparse hyphae; strains H4, H5, H7, H8, H10, and H12 had dense hyphae; and strain H6 had relatively dense hyphae. Based on a comprehensive analysis of the growth vigor of different *H. erinaceus* strains, strains H4, H9, and H10 were rated as excellent in growth vigor; strains H2, H3, H5, H8, and H12 were rated as good; and strains H1, H6, H7, and H11 were rated as average.

Table S4 Mycelial growth rate and growth vigor of *Hericium erinaceus* in conventional cottonseed hull medium

Strains	Growth rate（mm/d）	Color	Marginal regularity	Density	Growth
H1	1.99±0.09 b	white	relatively neat	sparse	+
H2	1.87±0.16 b	white	neat	sparse	++
H3	1.22±0.13 c	snow-white	neat	sparse	++
H4	2.6±0.12 a	snow-white	neat	dense	+++
H5	1.43±0.05 c	ashen	neat	dense	++
H6	1.87±0.11 b	snow-white	untidy	relatively dense	+
H7	1.88±0.12 b	ashen	untidy	dense	+
H8	1.9±0.14 b	snow-white	relatively neat	dense	++

H9	2.37 ± 0.11^{a}	snow-white	neat	bushy	+++
H10	2.57 ± 0.09^{a}	snow-white	neat	dense	+++
H11	1.77 ± 0.02^{b}	ashen	neat	sparse	+
H12	1.99 ± 0.16^{b}	ashen	neat	dense	++

Note: “+” poor growth; “++” growth is average; “+++” the growth is better than average；Different lowercase letters indicate significant differences, $p<0.05$

1.3 Strain Screening

Based on a comprehensive analysis of the mycelial growth rate and growth vigor of different strains on PDA medium and conventional cotton seed hull medium, the elite strains were eventually screened out: elite *P. geesteranus* strains included X2, X3, X4, X5, and X6; elite *H. erinaceus* strains included H4, H9, and H10. These strains were used for subsequent experiments.
